# GRAMD4 inhibits tumour metastasis by recruiting the E3 ligase ITCH to target TAK1 for degradation in hepatocellular carcinoma

**DOI:** 10.1002/ctm2.635

**Published:** 2021-11-17

**Authors:** Qian yun Ge, Jin Chen, Gan xun Li, Xiao long Tan, Jia Song, Deng Ning, Jie Mo, Peng cheng Du, Qiu meng Liu, Hui fang Liang, Ze yang Ding, Xue wu Zhang, Bi xiang Zhang

**Affiliations:** ^1^ Hepatic Surgery Center Tongji Hospital Tongji Medical College Huazhong University of Science and Technology Wuhan P. R. China; ^2^ Clinical Medical Research Center of Hepatic Surgery Wuhan P. R. China; ^3^ Hubei Key Laboratory of Hepato‐Pancreato‐Biliary Diseases Wuhan P. R. China; ^4^ Key Laboratory of Organ Transplantation Ministry of Education Wuhan P. R. China; ^5^ Key Laboratory of Organ Transplantation National Health Commission Wuhan P. R. China; ^6^ Key Laboratory of Organ Transplantation Chinese Academy of Medical Sciences Wuhan P. R. China

**Keywords:** GRAMD4, HCC, Metastasis, TAK1, Ubiquitination

## Abstract

**Background:**

Aberrant TAK1 (transforming growth factor β‐activated kinase 1) activity is known to be involved in a variety of malignancies, but the regulatory mechanisms of TAK1 remain poorly understood. GRAMD4 (glucosyltransferase Rab‐like GTPase activator and myotubularin domain containing 4) is a newly discovered p53‐independent proapoptotic protein with an unclear role in HCC (hepatocellular carcinoma).

**Results:**

In this research, we found that GRAMD4 expression was lower in HCC samples, and its downregulation predicted worse prognosis for patients after surgical resection. Functionally, GRAMD4 inhibited HCC migration, invasion and metastasis. Mechanistically, GRAMD4 interacted with TAK1 to promote its protein degradation, thus, resulting in the inactivation of MAPK (Mitogen‐activated protein kinase) and NF‐κB pathways. Furthermore, GRAMD4 was proved to recruit ITCH (itchy E3 ubiquitin protein ligase) to promote the ubiquitination of TAK1. Moreover, high expression of TAK1 was correlated with low expression of GRAMD4 in HCC patients.

**Conclusions:**

GRAMD4 inhibits the migration and metastasis of HCC, mainly by recruiting ITCH to promote the degradation of TAK1, which leads to the inactivation of MAPK and NF‐κB signalling pathways.

## Graphical abstract

We revealed a novel mechanism that GRAMD4 promotes the TAK1 ubiquitination and degradation by recruitment of ITCH, a E3 ubiquitin ligase of TAK1, which lead to the inhibition of MAPK and NF‐κB pathways and the downstream MMPs expression.

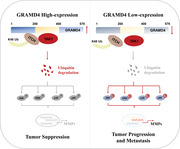

## 1 INTRODUCTION

As the sixth most common neoplasm and the third leading cause of cancer‐related deaths, hepatocellular carcinoma (HCC) accounts for 75‐85% of primary liver cancer cases.^1^, ^2^ The high mortality rate of HCC results from advanced stage at initial diagnosis, high incidence of tumour metastasis and recurrence of the tumour after surgical resection.^3^ In spite of significant progress over the past decades, patients with late‐stage HCC still have a poor prognosis. Therefore, a better understanding of the underlying molecular mechanisms driving the pathogenesis and metastasis of HCC is urgently needed.

GRAMD4 was first identified as a target gene of E2F1. It contains a nuclear localization signal, two transmembrane regions and a GRAM domain.^4^ It was reported that GRAMD4 interacts directly with Bcl‐2 to promote the delocalization of Bax in mitochondria and its oligomerization, leading to the induction of apoptosis.^4^, ^5^ GRAMD4 is associated with apoptosis in H1299, Saos‐2 cells and HCT 116 cells.^5^ However, there are few studies on the role of GRAMD4 in tumours. According to previous studies, the expression of GRAMD4 was elevated in lung squamous cell carcinoma and high GRAMD4 expression predicted poor clinical outcomes.^6^ It was also reported that overexpression of GRAMD4 resulted in the inhibition of TLR9 (toll‐like receptor 9)‐ and TLR3‐mediated immune responses.^7^ Nevertheless, much less is known about the role of GRAMD4 in HCC, and this study focused on the role of GRAMD4 in HCC metastasis and the underlying mechanisms.

Tumour progression and metastasis are strongly associated with TAK1 (transforming growth factor β‐activated kinase 1) activity, and TAK1 deficiency leads to the deactivation of NF‐κB subunit p65, resulting in enhanced chemosensitivity in HCC cells and reduced tumourigenesis in lung cancer.^8^, ^9^ Transcriptional upregulation of multiple metastasis‐related genes, including chemokine receptors and matrix metalloproteinases (MMP), is induced by increased TAK1 activity.^10^ For example, inhibition of TAK1 expression was found to reduce the expression of MMP9 and the invasive capacity of breast cancer and gastric cancer.^11^, ^12^

TAK1 is a key intermediate that transmits signals from receptor complexes to MAPKs and IKK (inhibitor of NF‐κB (IκB) kinase).^13^ Preventing the overactivation of TAK1 is important for inhibiting the progression of cancer and inflammatory diseases. Clusterin inhibits TGFBR1 (transforming growth factor‐β receptor) from recruiting the TRAF6 (TNF receptor‐associated factor 6)/TAB2 (TAK1‐binding protein 2)/TAK1 complex, which leads to the activation of the TAK1/NF‐κB signalling pathways to block the progression of lung cancer.^8^ DUSP14 (dual‐specificity phosphatase 14) can ameliorate the key pathological processes and inflammatory responses related to the development of non‐alcoholic fatty liver disease by associating with and dephosphorylating TAK1, which in turn leads to reduced activation of TAK1 and its downstream signalling molecules such as NF‐κB, JNK, ERK and p38.^14^ In addition, TAK1 degradation induced by polyubiquitination at Lys48 also plays a critical role in the inactivation of multiple signals controlled by TAK1.^15^ Recently, ITCH was proposed to act as a E3 ligase of TAK1 that mediates Lys48 polyubiquitination and negatively regulates NF‐κB activation induced by TNF‐α (tumour necrosis factor alpha).^16^

In this study, we revealed a novel mechanism in which GRAMD4 inhibits the invasion and metastasis of HCC by recruiting ITCH to promote TAK1 ubiquitination at Lys48. Our results indicate that GRAMD4 was downregulated in clinical HCC tissues and negatively correlated with TAK1. Furthermore, the downregulation of GRAMD4 predicted poor clinical outcomes in HCC patients. Functional assays demonstrated that GRAMD4 suppressed the migration, invasion and motility of HCC cells in vitro and repressed HCC metastasis in vivo. Moreover, we verified that GRAMD4‐mediated TAK1 downregulation is critical for the suppression of the invasion and metastasis of HCC.

## 2 MATERIALS AND METHODS

### 2.1 Patients and HCC tissue specimens

A cohort of 50 pairs of HCC tissues and peritumour normal tissues and another cohort of 110 pairs of paraffin‐embedded peritumour normal liver tissues and HCC tissues were obtained from HCC patients who underwent curative resection between 2012 and 2014 at the Hepatic Surgery Center, Tongji Hospital. All the patients understood the procedure and provided written consent for the study. This study was approved by the Ethics Committee of Tongji Hospital.

### 2.2 Gene expression datasets

For the TCGA cohort, data, including the RNA‐seq profiles of HCC patients, were downloaded from the TCGA database (https://tcga‐data.nci.nih.gov/tcga/). This cohort contained 362 HCC patients with GRAMD4‐expression data, with corresponding follow‐up information. The TCGA cohort was used to explore the copy‐number variation (CNV) levels of GRAMD4 in HCC. In addition, three sets of mRNA microarray data were obtained from the GEO database (http://www.ncbi.nlm.nih.gov/geo/), including GSE14520, GSE22058 and GSE63898. These data were used to validate GRAMD4 expression levels in HCC. All Proteomics data were downloaded from the National Cancer Institute Clinical Proteomic Tumor Analysis Consortium (CPTAC) Data Portal (https://cptac‐data‐portal.georgetown.edu/cptacPublic/)^17^

### 2.3 Cell culture

Human hepatocellular carcinoma cell lines HLF, Huh7, Hep3B, HepG2 and human embryonic kidney cell line HEK293 were obtained from China Center for Type Culture Collection. HCC cell lines MHCC97‐H and HCC‐LM3 were obtained from the Liver Cancer Institute, Zhongshan Hospital, Fudan University, Shanghai, China. These cells were culture in DMEM (Gibco, USA) supplemented with 10% foetal bovine serum (Gibco) in a humidified atmosphere comprising 5% CO_2_ at 37°C.

### 2.4 Plasmids and lentivirus

A HLF cell line that stably overexpressed GRAMD4 was constructed by introducing a vector encoding the Flag‐tagged CDS of GRAMD4 cloned into the pLenti‐CMV‐Puro plasmid (Addgene #17448).

The shRNAs targeting the specific genes and negative controls were cloned into the plasmid pLKO.1‐TRC (Addgene #10879). The coding sequences of human GRAMD4 and the control sequences were sub‐cloned into the pLenti‐CMV‐Puro plasmid (Addgene #17448). The recombinant vectors encoding Flag‐, HA‐, or Myc‐tagged GRAMD4, TAK1, ITCH and the mutants were sub‐cloned into the pcDNA3.1(‐) plasmid. The packaging plasmids pMD2.G (Addgene#12259), psPAX2 (Addgene #12260) and pLenti‐CMV‐Puro or pLKO.1‐shRNA plasmids (Ratio 1:3:4) were used for virus packaging. Collected virion‐containing supernatants were used to infect HLF and Hep3B cells for 48 h, after which stably transduced cells were selected by cultured in medium containing 2.5 μg/mL puromycin for 2 weeks. The target sequences for specific genes were as follows: shGRAMD4‐1: 5′‐AGCACCAAGAAGGGCAATTTC‐3′; shGRAMD4‐2: 5′‐GCCATCCCATTGTTCTTATTT‐3′; shGRAMD4‐3: 5′‐GAGATCTTCAATCTGACAGAA‐3′; ShTAK1: GCAGTGATTCTTGGATTGTTT.

### 2.5 Antibodies and reagents

Primary antibodies against GRAMD4 (24299‐1‐AP for WB, immunohistochemistry (IHC), immunofluorescence (IF) and (IP) and ITCH (20920‐1‐AP for WB, IP) were purchased from Proteintech (Wuhan, Hubei, China); primary antibodies against TAK1 (#5206 for WB, IHC, IF and IP), ERK (#4695 for WB), phospho‐ERK (#4370 for WB), p38 ((#8690 for WB), phospho‐p38 (#9215 for WB), JNK (#9252 for WB), phospho‐JNK(#9255 for WB) and Myc‐tag (#2276 for WB) were from CST (Danvers, MA, USA); Mouse serum IgG (I5381 for IP control), Rabbit serum IgG (I5006 for IP control), primary antibodies against Flag‐tag (F1804 for WB, IP and IF) and HA‐tag (H6908 for WB, IP and IF) were from Sigma (St. Louis, MO, USA); HRP‐conjugated anti‐Rabbit IgG (#111‐035‐003) and HRP‐conjugated anti‐Mouse IgG (#115‐035‐003) secondary antibodies were used for WB analysis. Laboratories (PA, USA). HRP‐conjugated anti‐Rabbit IgG Light Chain (A25022 for WB) and HRP‐conjugated anti‐Mouse IgG Light‐Chain (A25012 for WB) secondary antibodies were purchased from Abbkine (California, USA). Fluorochrome‐conjugated secondary antibodies were from Thermo Fisher Scientific (USA). Cycloheximide and MG132 were purchased from Calbiochem (San Diego, CA, USA).

### 2.6 IF and IHC analyses

IF assays were performed as described previously.^18^ GRAMD4 and TAK1 antibodies were diluted to 1: 100. IHC analysis was performed according to a previously reported protocol.^19^ The immunohistochemical staining was scored according to the percentage of positively stained tumour cells and staining intensity score. Positively stained cells were scored as follows: less than 10% scored as 0, 10–25% scored as 1, 26–50% scored as 2, 51–75% scored as 3 and more than 75% positively stained tumour cells was scored as 4. In addition, the staining intensity was evaluated as follows: the score scale was 0 to 3, negative for 0, light brown for 1, brown for 2 and dark brown for 3. The total scores were obtained by multiplying the above two, 0–5 were defined as negative and 6–12 were defined as positive.

### 2.7 Immunoblotting and immunoprecipitation

Immunoblotting and co‐immunoprecipitation (co‐IP) experiments were performed as described previously.^20^ Cells were harvested and total cell lysates were obtained using IP‐lysis buffer (protease inhibitor cocktail, 10% glycerol, 1 mM EDTA l, 1% Triton X‐100, 150 mM NaCl and 50 mM Tris‐HCl, pH7.4) with phosphatase inhibitor and protease inhibitor (Thermo). Total lysates were then fractionated by SDS‐PAGE and transferred to PVDF membranes. After blocking with 5% skim milk, the membranes were incubated with primary antibodies overnight at 4°C. Then, the blots were washed three times with Tris Buffered Saline + Tween (TBST) and reacted with the HRP‐conjugated mouse and rabbit secondary antibodies for 1 h at room temperature. The immunoreactive bands were analysed using ImageJ software.

### 2.8 Quantitative real‐time PCR assay

Trizol reagent (Invitrogen, , Carlsbad, CA, USA) was used to extract the total RNA from harvested cells. The total RNA was reverse‐transcribed using the RT‐PCR kit (Promega) to obtain the cDNA. For real‐time PCR, the cDNA was amplified with specific primers using a SYBR Green PCR Kit (Qiagen). The primers used are summarized in Supporting information Table S1.

### 2.9 Wound healing and transwell migration assays

HFL and Hep3B cells were seeded into the top chambers of a 24‐well transwell plate (Corning) with or without Matrigel (BD Biosciences) for invasion or migration assays according to the Product Specification Sheet. The cells were harvested after 24 h. Cells on the bottom of the upper chamber were fixed and stained. The migrated or invading cells were scored in 10 randomly chosen microscopic fields under an optical microscope. For the wound healing assay, HLF and Hep3B cells were cultured in DMEM without foetal bovine serum overnight, after which a pipette tip was used to introduce a scratch. Photographs of five random fields were captured by phase contrast microscopy at 0, 24 and 48 h after the wounding. Each experiment was repeated at least three times.

### 2.10 RNA interference

Lipofectamine 3000 (Invitrogen) was used for siRNA transfection. After transfection for 48 h at 37℃, cells were harvested and analysed by western blotting. The siRNAs for the specific targets and si‐control were purchased from RiboBio (Guangzhou, China). The siRNA targeting TAK1 was as follows: si‐TAK1: 5‐ CGGAACCTTTAGGGATAGTTC ‐3; the siRNA targeting ITCH was as follows: si‐ITCH: 5‐ GAAGAACTACCACCTATAT ‐3

### 2.11 Lung metastasis assay in vivo

Female BALB/C nude mice aged 5 weeks were used for this assay. A total of two mouse models were used to study in vivo metastasis, with each group composed of five mice. For the lung metastasis models, 1.5 × 10^6^ of the indicated HLF or Hep3B HCC cells were re‐suspended in serum‐free medium and injected into the tail vein of each mouse. After 8 weeks, the mice were sacrificed and their lung tissues were collected, measured and photographed. Subsequently, the lung tissues were fixed with 4% formalin for 72 h and embedded in paraffin for haematoxylin and eosin (H&E) staining. For orthotopic models, HCC cells (1 × 10^6^) in 30 μL serum‐free DMEM were injected into the left hepatic lobe of nude mice. Each group was composed of six mice. After 8 weeks following implantation, the mice were sacrificed and visible tumour nodules in the livers were counted. All animal experiments were carried out in compliance with the guidelines of the Animal Care Committee and were approved by the Ethics Committee of Tongji Hospital of HUST.

### 2.12 Statistical analyses

Statistical analyses were conducted in SPSS 22.0 software (IBM Corp., USA) or GraphPad Prism 5 software (GraphPad, CA, USA). Student's *t*‐test was used to compare continuous variables that conform to a normal distribution, and the Mann–Whitney U test was used to compare variables that do not conform to a normal distribution. Pearson's χ^2^ or Fisher's exact test were used to analyse categorical variables. The Kaplan–Meier method was used for the survival analysis. Pearson's correlation test was used to evaluate correlations for continuous variables. A *p*‐value less than 0.05 was considered to indicate statistical significance, and the data were presented as mean ± SEM or mean ± SD.

## 3 RESULTS

### 3.1 GRAMD4 expression was downregulated in HCC and this downregulation was correlated with a poor prognosis

**FIGURE 1:**
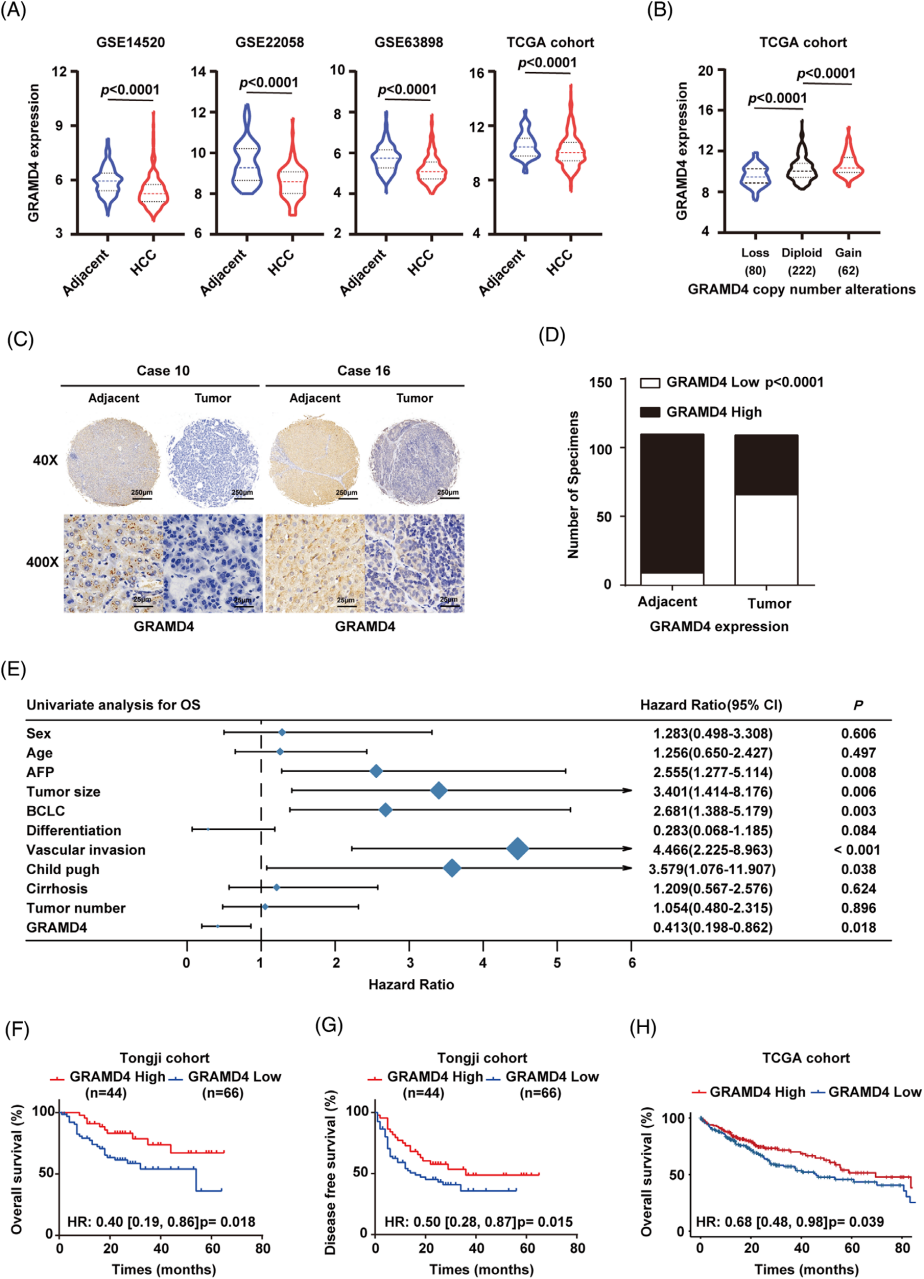
GRAMD4 expression was downregulated in HCC and predicted poor clinical outcomes. (A) GRAMD4 mRNA expression in HCC tissues and adjacent normal samples extracted from GSE22058, GSE14520, GSE63898 and TCGA. (B) GRAMD4 CNV status in HCC from TCGA. (C) Representative immunohistochemical staining images of GRAMD4 in 110 paired HCC tumour tissues and adjacent non‐tumour tissues (Scale bar: 250 μm, 25 μm). (D) Statistical analysis of GRAMD4 expression according to the IHC assay in HCC tissues and matched adjacent tissues. (E) Univariate regression analysis of the relation between the GRAMD4 and clinicopathological characteristics regarding OS in the Tongji cohort. Overall survival (F) and disease‐free survival (G) of HCC patients with low or high tumour GRAMD4 scoring in the Tongji cohort. Overall survival (H) of HCC patients with low or high GRAMD4 expression in the TCGA cohort

To determine the role of GRAMD4 in HCC, we examined GRAMD4 levels in tumour samples and paired normal tissues from the GSE22058,^21^ GSE14520,^22^ GSE63898^23^ datasets and TCGA. The results indicated that GRAMD4 expression was downregulated in HCC tissues (Figure 1A). To understand what might govern the downregulation of GRAMD4 in HCC, we next examined potential factors regulating its expression. According to our previous research, there were no significant differences in promoter methylation of GRAMD4 in HCC (Supporting information Figure S1).^24^ However, we noted that the reduction of GRAMD4 mRNA expression in HCC was mainly caused by the loss of GRAMD4 due to CNV in the TCGA cohort (Figure 1B). In addition, we also explored the signalling pathways affected by the downregulation of GRAMD4 in HCC tissues from TCGA database, and the results revealed that the E2F, ATR and VEGF pathways might play a role in the downregulation of GRAMD4 in HCC (Supporting information Figure S2A). To confirm the decreased GRAMD4 expression in HCC, we performed IHC in a HCC tissue microarray from the Tongji cohort. IHC staining and scoring showed that the expression of GRAMD4 was lower in HCC tissues (Figure 1C and D). We further explored whether GRAMD4 is an independent prognostic factor for HCC patients in the Tongji cohort using univariate and multivariate Cox regression analyses. After adjusting for the conventional clinical patterns, including sex, age at diagnosis, Child‐Pugh score, serum AFP level, cirrhosis, number of lesions, BCLC stage, vascular invasion, tumour differentiation and tumour size, the results revealed that GRAMD4 expression had a strong predictive ability for the OS of HCC patients (OS: HR, 0.40; 95% CI:, 0.19–0.86; *p*  = 0.018, Figure 1E, Supporting information Figure S2B). Furthermore, we also confirmed that GRAMD4 was an independent predictive factor for the DFS of HCC patients in the Tongji cohort (HR, 0.496; 95% CI:, 0.282–0.873; *p*  = 0.015, Supporting information Figure S2C and D). Subsequently, we used Kaplan–Meier survival analysis to explore the effects of GRAMD4 on the OS of HCC patients, and the results revealed that HCC patients with relatively lower GRAMD4 expression had shorter OS in both the Tongji and TCGA cohorts (Figure 1F and H), as well as shorter DFS in the Tongji cohort (Figure 1G). In general, these data indicated that GRAMD4 was downregulated in HCC and the decreased GRAMD4 expression predicted poor clinical outcomes in HCC patients.

### 3.2 GRAMD4 acts as a suppressor of HCC metastasis in vitro and in vivo

**FIGURE 2:**
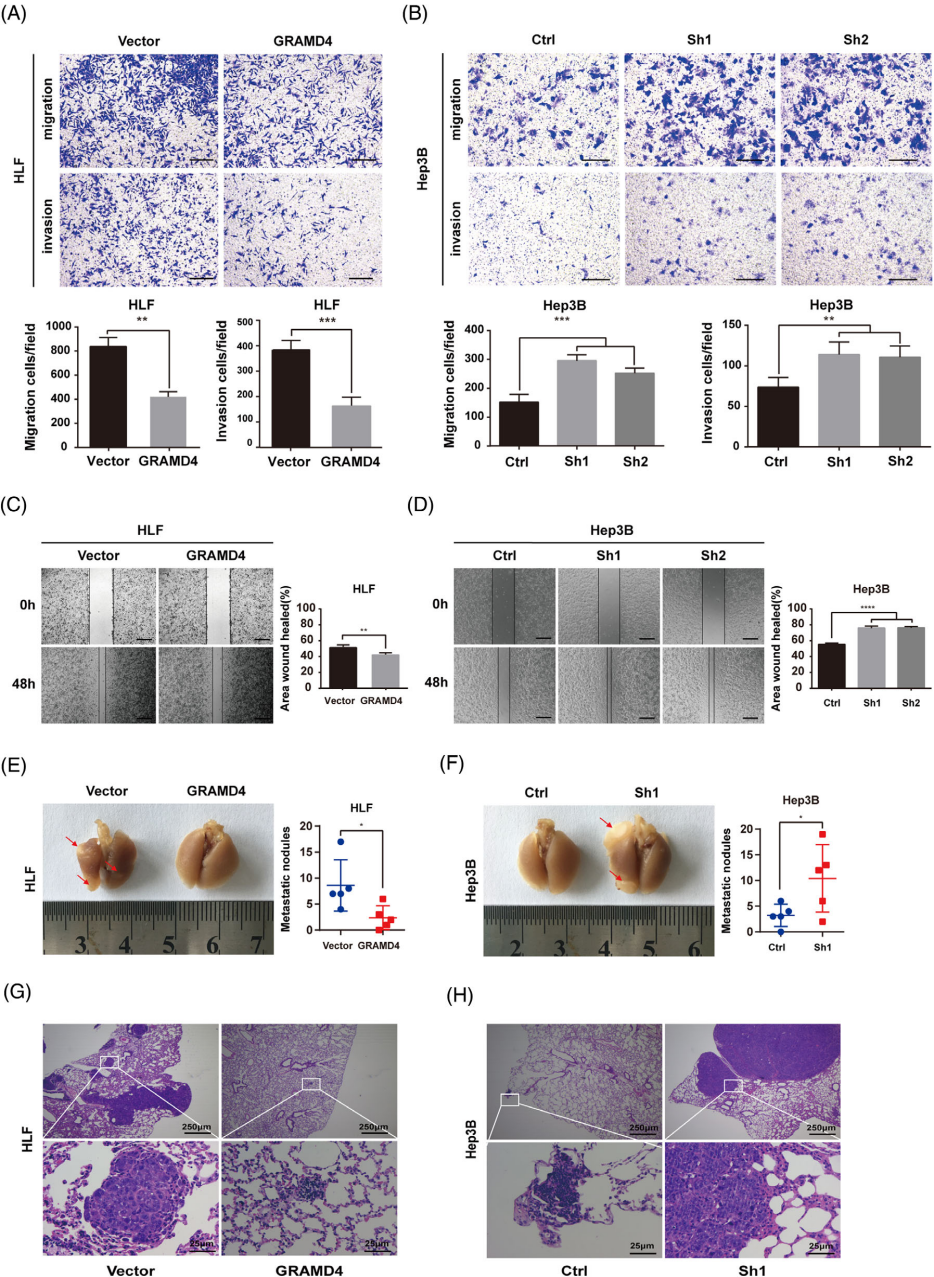
GRAMD4 suppresses metastasis of HCC in vitro and in vivo. (A) Trans‐well migration and invasion assays were performed using HLF‐Vector and HLF‐GRAMD4 stably transformed cells. (B) Trans‐well migration and invasion assays were performed with Hep3B‐Ctrl, Hep3B‐Sh1 and Hep3B‐Sh2 stably transfected cells. Wound healing assays were performed with HLF‐GRAMD4 (C) and Hep3B‐shGRAMD4 (D) stably transfected cells. Representative images are shown. Experiments were performed in triplicates and the data are shown as mean ± SD. Scale bar, 100 μm. Lung metastasis experiments were conducted in nude mice with HLF‐GRAMD4 (E, G) and Hep3B‐shGRAMD4 (F, H) stably transfected cells. Representative images of lung metastases and H&E staining of lung tissues are shown. The data represent mean ± SD. Statistical analysis was performed using Student's unpaired *t*‐test in (A‐D). *p < 0.05, **p < 0.01, ***p < 0.001, ****p < 0.0001

We next explored the roles of GRAMD4 in HCC progression by evaluating cell migration and metastasis. Based on the expression of GRAMD4 in the seven tested HCC cell lines (Supporting information Figure S3A), we decided to investigate whether GRAMD4 had an influence on the malignant phenotypes of HCC cells. Three small hairpin RNAs specifically targeting GRAMD4 were used to stably transfect Hep3B cells, resulting in the cell lines Hep3B GRAMD4‐Sh1, Hep3B GRAMD4‐Sh2 and Hep3B GRAMD4‐Sh3. Additionally, a scrambled shRNA was also included as a negative control, resulting in the cell line Hep3B‐Ctrl. Among these cell lines, Hep3B GRAMD4‐Sh1 and Hep3B GRAMD4‐Sh2 with the highest knockdown efficiency were selected for further study. Moreover, HLF cells were transfected with a lentiviral GRAMD4 expression vector, with an empty vector as control, resulting in strains HLF‐GRAMD4 and HLF‐Vector, respectively. Western blot assays were used to validate the efficiency of GRAMD4 overexpression and knockdown (Supporting information Figure S3B and C). The transwell assay indicated that GRAMD4 overexpression obviously inhibited the migration and invasion ability of HLF cells (Figure 2A). Conversely, the depletion of GRAMD4 enhanced the migration and invasion ability of Hep3B cells (Figure 2B). Furthermore, the wound healing assay also indicated that migration was slower in HLF cells with GRAMD4 overexpression than in control cells (Figure 2C), while the healing rate was increased by the downregulation of GRAMD4 in Hep3B cells (Figure 2D). However, GRAMD4 had no effects on the proliferation of HCC cells (Supporting information Figure S3D‐G).

To further explore the effects of GRAMD4 on tumour growth and metastasis in vivo, we used a tail vein injection mouse model. HLF‐Vector, HLF‐GRAMD4, Hep3B‐Ctrl, or Hep3B‐Sh1 cells were injected into the tail vein of immunocompromised nude mice, respectively, and the mice were sacrificed 8 weeks later. The results of histopathological analysis showed that GRAMD4 overexpression attenuated the lung metastasis of HLF cells (Figure 2E and G). Conversely, downregulation of GRAMD4 increased the lung metastasis of Hep3B cells (Figure 2F and H). Furthermore, the orthotropic model suggested that the GRAMD4 overexpression group had less and smaller liver metastatic nodules than the vector group, while downregulation of GRAMD4 led to more and larger liver metastatic nodules (Supporting information Figure S3H‐K). These data suggested that GRAMD4 acts as a tumour suppressor and inhibits the migration, invasion and lung metastasis of HCC cells.

### 3.3 GRAMD4 interacted and co‐localized with TAK1

**FIGURE 3:**
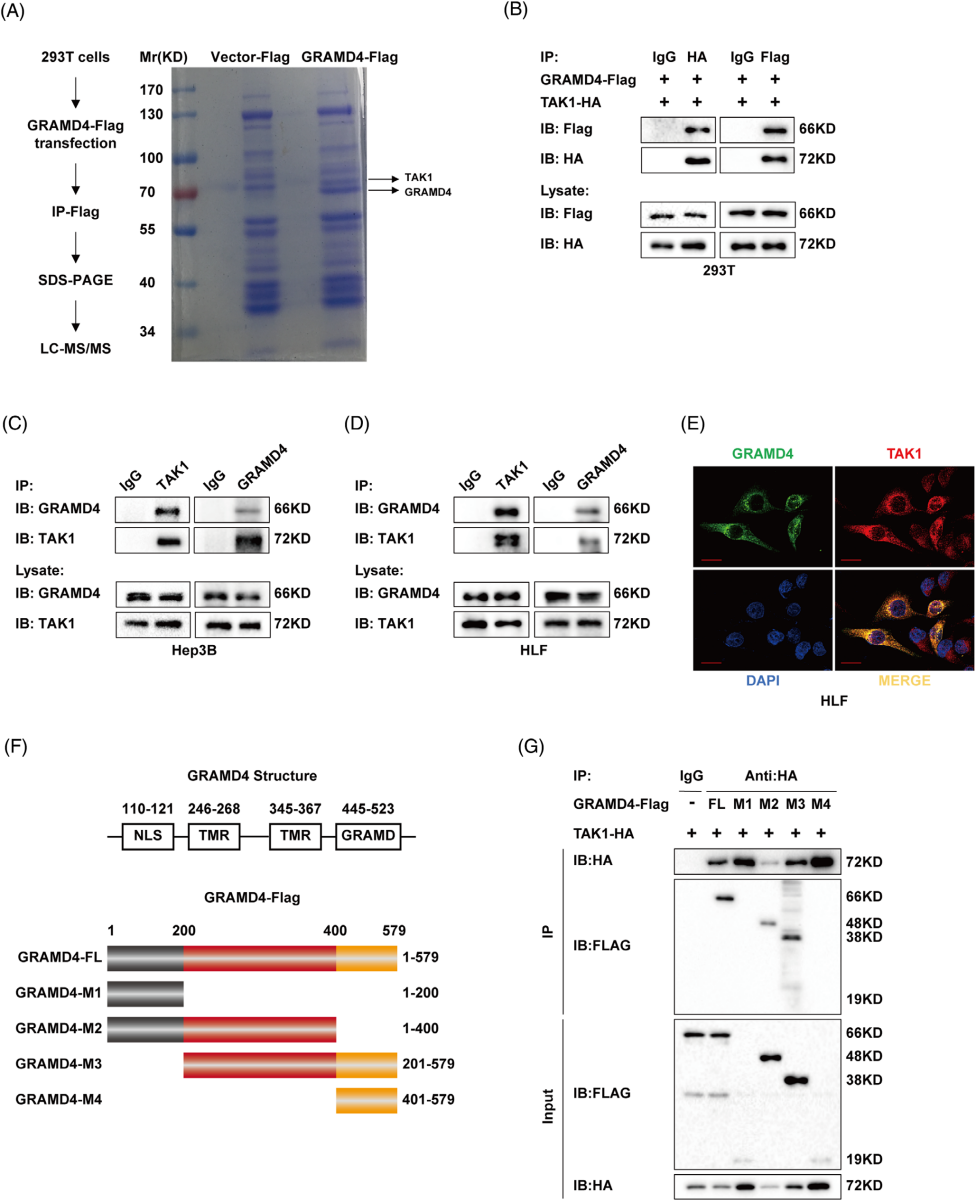
GRAMD4 interacted and co‐localized with TAK1. (A) MS analysis of GRAMD4‐associated proteins. (B) Co‐IP analysis of the binding between exogenous GRAMD4‐Flag and TAK1‐HA in co‐transfected HEK293T cells. (C‐D) Co‐IP analysis of the binding between endogenous GRAMD4 and TAK1 in HLF and Hep3B cells. (E) HLF cells were fixed and stained with TAK1 antibody (Red) and GRAMD4 antibody (Green). Nuclei were counterstained with DAPI (blue). Scale bar: 20μm. (F) Schematic illustration showing the wild‐type and truncation mutants of GRAMD4. (G) Co‐IP analysis of the interaction between TAK1 and full‐length GRAMD4 or its truncation mutants in HEK293T cells co‐transfected with TAK1‐HA plasmid and GRAMD4‐Flag plasmid or GRAMD4‐Flag truncation mutant plasmids

To identify the mechanism underlying the inhibitory effects of GRAMD4 in HCC, we used a combined IP/MS approach to identify novel GRAMD4‐mediated protein–protein interactions. The total cell extracts prepared from HEK293T cells overexpressing FLAG‐tag or FLAG‐tagged GRAMD4 were immunoprecipitated and analysed by gradient elution LC‐MS/MS (Figure 3A). Differentially expressed proteins between the two types of immunoprecipitated lysates were identified based on at least two peptides with ≥95% confidence with an averaged ratio‐fold change ≥2 in the Flag group versus the IgG group. According to these criteria, a total of 32 differentially expressed proteins were found (Supporting information Table S2). MAP3K, an intracellular serine/threonine kinase that was reported to be associated with tumour progression,^25^ ranked third (Supporting information Figure S4A). To confirm the MS data, flag‐tagged GRAMD4 and HA‐tagged TAK1 were co‐expressed in HEK293T cells, and the co‐IP results indicated that these two exogenous proteins interact (Figure 3B). Moreover, endogenous GRAMD4 also bound to endogenous TAK1 in HLF and Hep3B cells (Figure 3C and D). Furthermore, confocal immunofluorescence microscopy revealed a high degree of spatial concordance between endogenous GRAMD4 and TAK1 in HLF and Hep3B cells (Figure 3E, Supporting information Figure S4B). These results showed that GRAMD4 interacts with TAK1. To identify the region of GRAMD4 responsible for the interaction with TAK1, we generated a series of domain‐truncation mutants of GRAMD4 (Figure 3F). In the *N‐*terminal region of GRAMD4, there was a nuclear localization signal, and the *C*‐ terminal region contained a GRAM domain, which is often found in membrane‐associated proteins.^4^ Moreover, two transmembrane regions were found, spanning the amino acids 246–268 and 345–367, respectively (Figure 3F). Our results showed that the amino acids 200–400 of GRAMD4 (encompassing two transmembrane regions) were required for its interaction with TAK1 (Figure 3G). Taken together, these data demonstrated that GRAMD4 could interact with TAK1, and the interaction was mediated by the transmembrane regions of GRAMD4.

### 3.4 The anti‐tumour effect of GRAMD4 is mediated by its effect on TAK1 protein levels

**FIGURE 4:**
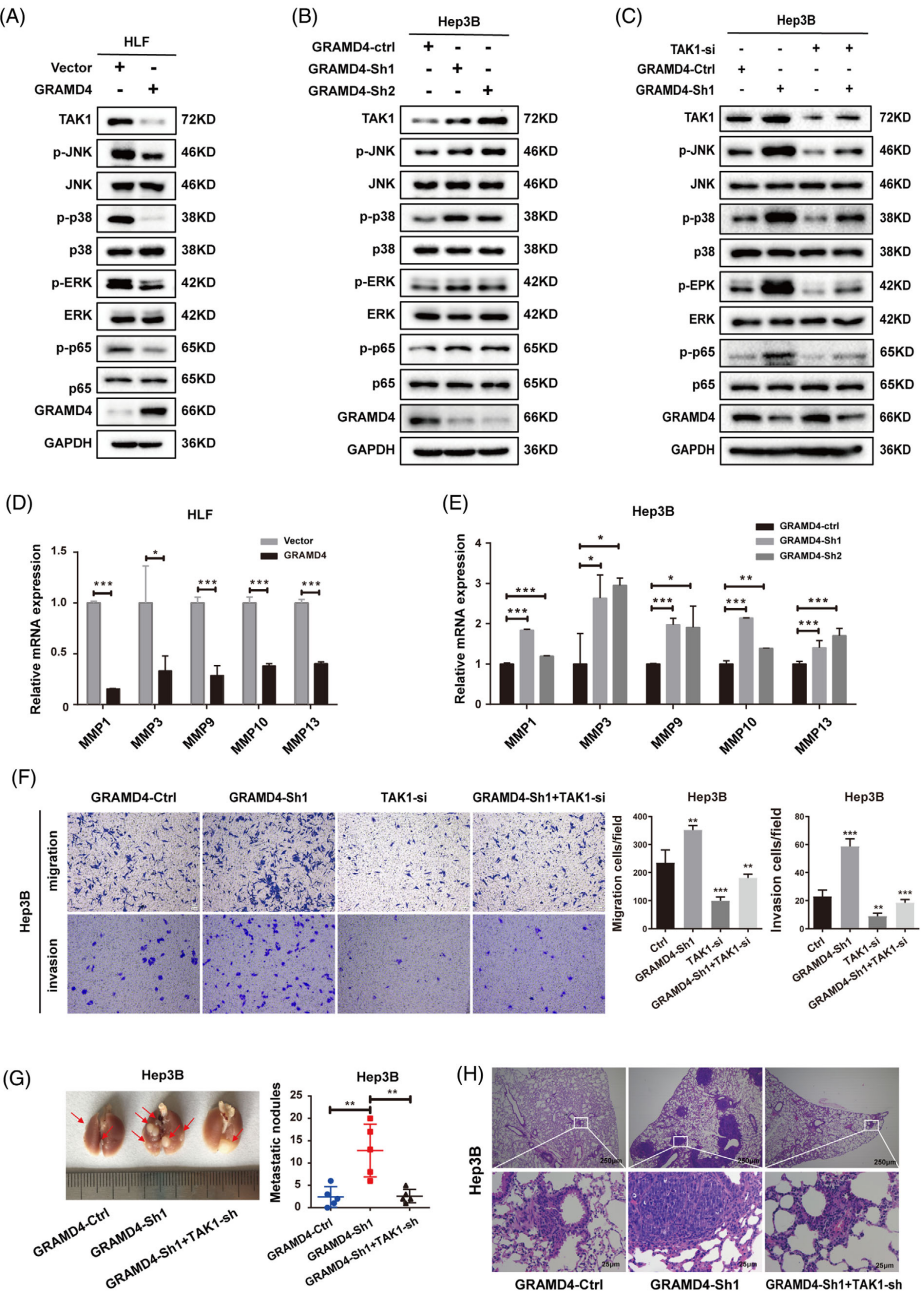
GRAMD4 exerted its tumour suppressive effects by modulating the protein levels of TAK1. (A) Western blot analysis of TAK1, p‐JNK, p‐p38, p‐ERK and p‐p65 protein levels in HLF cells stably transfected with GRAMD4 plasmid or vector control. (B) Western blot analysis of TAK1, p‐JNK, p‐p38, p‐ERK, and p‐65 protein levels in Hep3B cells stably transfected with lentivirus expressing Sh‐Ctrl, GRAMD4‐Sh1, or GRAMD4‐Sh2. (C) Western blot analysis of TAK1, p‐JNK, p‐p38, p‐ERK and p‐65 protein levels in GRAMD4‐depleted Hep3B cells transiently transfected with si‐NC, TAK1‐si. (D, E) The expression levels of MMP‐1, MMP‐3, MMP‐9, MMP‐10 and MMP‐13 in the indicated cells were analysed by qRT‐PCR. Experiments were performed in triplicate and data are shown as mean ± SD. (F) Transwell migration and invasion assays were performed with stably transfected HLF‐vector and HLF‐GRAMD4 cells. (G, H) Lung metastasis experiments were conducted in nude mice with the indicated stably transfected cells. Representative images of lung metastases (G) and H&E staining of lung tissues (H) are shown. Statistical analysis was performed using Student's unpaired *t*‐test in (F), and the Mann–Whitney U test in (G). *p < 0.05, **p < 0.01, ***p < 0.001, ****p < 0.0001

After identifying the protein–protein interaction between GRAMD4 and TAK1, we investigated its role in the inhibition of HCC progression by GRAMD4. We found that GRAMD4 overexpression reduced the protein level of TAK1 (Figure 4A), and downregulation of GRAMD4 increased the protein level of TAK1 (Figure 4B). However, the change of GRAMD4 expression had no obvious effects on the mRNA level of TAK1 (Supporting information Figure S5A and B). Since TAK1 is an important kinase of the NF‐κB and MAPK pathways, which plays crucial roles in cancer growth and metastasis,^26^, ^27^ we examined the phosphorylation of p65 and several MAPKs in cell lines with GRAMD4 overexpression or knockdown. GRAMD4 overexpression efficiently reduced the phosphorylation of JNK, p38, ERK and p65 in HLF cells (Figure 4A), while GRAMD4 downregulation enhanced the phosphorylation of JNK, p38, ERK and p65 (Figure 4B). These results indicated that GRAMD4 reduced the abundance of TAK1 and inhibited the activation of the NF‐κB and the MAPK pathways. Furthermore, we also explored the role of GRDM4 in HCC cells stimulated with biological ligands that activate MAPK or NF‐kB such as TNF‐α. The results revealed that GRAMD4 overexpression efficiently reduced the phosphorylation of JNK, p38, ERK and p65 in HLF cells both with and without TNF‐α stimulation (Supporting information Figure S5D), while GRAMD4 downregulation enhanced the phosphorylation of JNK, p38, ERK and p65 with and without stimulation (Supporting information Figure S5E).

We next investigated whether TAK1 is involved in the inhibitory effect of GRAMD4 on the MAPK pathway. Our data further showed that knockdown of TAK1 could rescue the GRAMD4 deficiency‐mediated upregulation of the NF‐κB and MAPK signalling pathways (Figure 4C). Hydrolysis of the extracellular matrix (ECM) is an important factor of cancer cell invasion and metastasis, and previous studies revealed that matrix metalloproteases (MMPs) could promote the degradation of the ECM.^28^ The expression of several MMPs was found to be regulated by a variety of transcriptional factors such as activator protein 1 (AP‐1) and specificity protein 1 (SP1).^29^, ^30^ According to our results, the expression levels of MMPs, including MMP1, MMP3, MMP9, MMP10 and MMP13, were decreased upon the upregulation of GRAMD4 (Figure 4D), and increased after GRAMD4 downregulation (Figure 4E). To further confirm whether TAK1 was required for the inhibitory effect of GRAMD4 on the expression of MMPs, we examined the mRNA levels of MMPs after depletion of TAK1 in GRAMD4‐knockdown Hep3B cells. The results indicated that the upregulation of MMPs following GRAMD4 knockdown could be rescued by the depletion of TAK1 (Supporting information Figure S5C). To identify whether GRAMD4‐mediated downregulation of TAK1 was responsible for its anti‐tumour effects, GRAMD4‐knockdown Hep3B cells were transfected with an siRNA against TAK1. The results revealed that the increase of migration, invasion and motility caused by GRAMD4 knockdown in Hep3B cells was reversed by TAK1 knockdown (Figure 4F, Supporting information Figure S5F). Additionally, we used a tail vein injection mouse model to evaluate the in vivo effects of TAK1 on HCC lung metastasis induced by GRAMD4 downregulation. GRAMD4‐knockdown Hep3B cells were transfected with an shRNA targeting TAK1 (Supporting information Figure S5G‐H) to modulate its expression, and the results indicated that TAK1 knockdown prevented the increase of lung metastasis induced by GRAMD4 downregulation (Figure 4G and H). Taken together, our data demonstrate that GRAMD4 acts as a tumour suppressor by inhibiting the TAK1/MAPK axis in HCC.

### 3.5 GRAMD4 facilitated the Lys48‐polyubiquitination and degradation of TAK1

**FIGURE 5:**
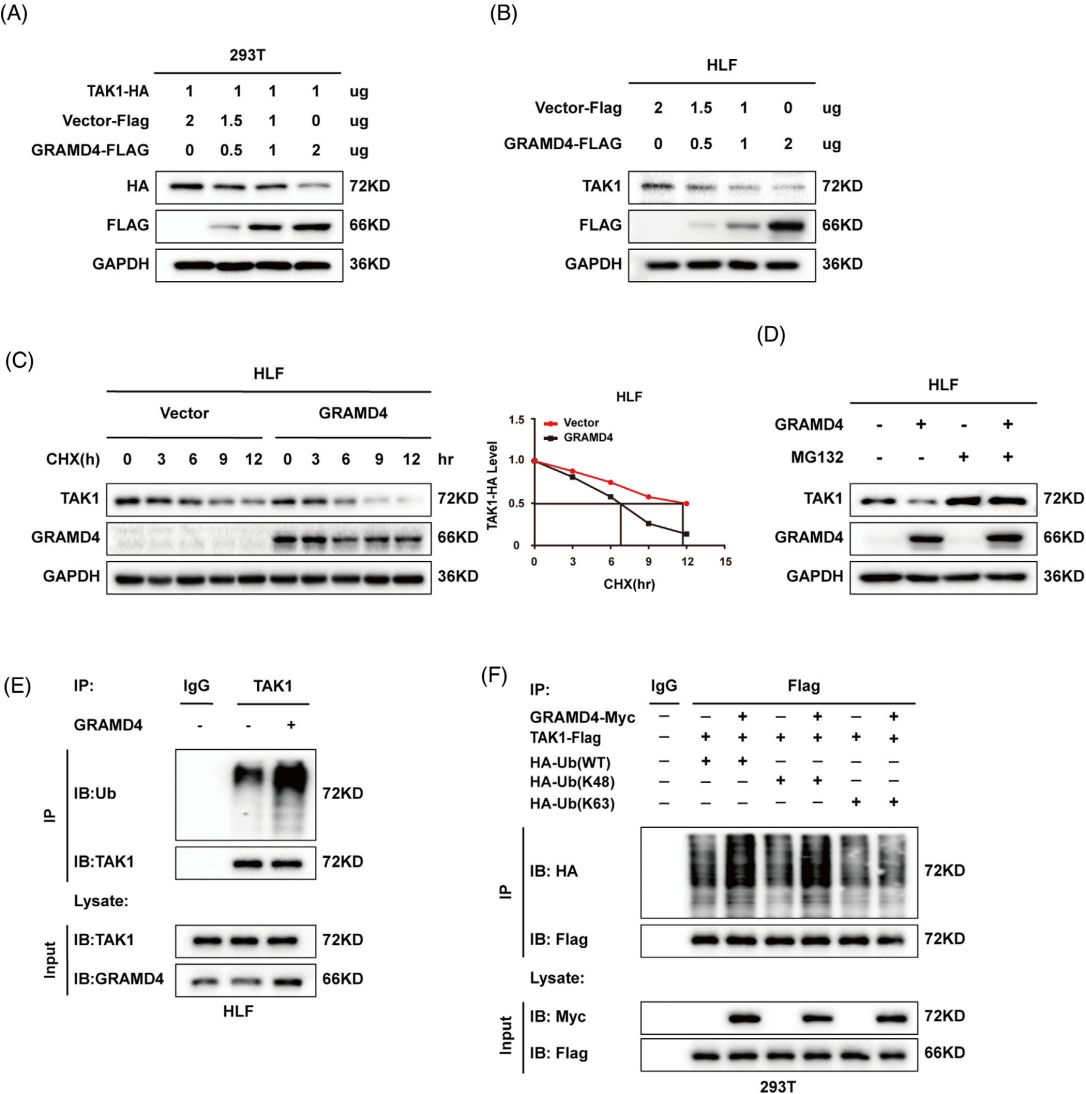
GRAMD4 facilitated the K48‐polyubiquitination‐dependent degradation of TAK1. (A) HEK293T cells were transfected with TAK1 (1 μg) and various concentrations of GRAMD4‐Flag plasmids. After 48 h, cell lysates were subjected to immunoblotting with anti‐Flag, anti‐HA and anti‐GAPDH antibodies. (B) HLF cells were transfected with various concentrations of GRAMD4‐Flag plasmid. After 48 h, cell lysates were subjected to immunoblotting with anti‐Flag, anti‐TAK1 and anti‐GAPDH antibodies. (C) HLF cells were transfected with GRAMD4 or vector control and were cultured for 24 h before being further incubated with CHX (20 μg/mL) for 0, 3, 6, 9 and 12 h. The TAK1 protein levels of the transfected cells were determined by western blot analysis. (D) HLF‐GRAMD4 or control cells were treated with MG132 (10 μM) for 6 h before immunoblot analysis for TAK1 and GRAMD4 levels. (E) HLF‐GRAMD4 or control cells were treated with Mg132 (10 μM) for 6 h, then TAK1 ubiquitination was measured via immunoprecipitation (IP) of TAK1. (F) Co‐IP analysis of ubiquitination of TAK1 in HEK293T cells co‐transfected with GRAMD4‐Myc plasmid, TAK1‐Flag plasmid and HA‐Ub‐K48, HA‐Ub‐WT, or HA‐Ub‐K63 plasmid

As mentioned above, GRAMD4 negatively regulated the protein abundance of TAK1, but had no effects on its mRNA level. Furthermore, expression of HA‐TAK1 together with increasing amounts of Flag‐GRAMD4 in HEK293T cells resulted in lower abundance of TAK1 (Figure 5A). Similarly, GRAMD4 inhibited the expression of TAK1 in a dose‐dependent manner in HLF cells (Figure 5B). The post‐transcriptional destabilization of TAK1 by GRAMD4 suggested that the latter might promote TAK1 protein degradation. As predicted, overexpression of GRAMD4 significantly reduced the half‐life of TAK1 after inhibiting protein synthesis with cycloheximide in HLF and HEK293T cells (Figure 5C, Supporting information Figure S6). Furthermore, to validate whether GRAMD4 leads to TAK1 degradation in a proteasome‐dependent manner, HLF cells with or without GRAMD4 overexpression were treated with the proteasome inhibitor MG132, which prevented TAK1 degradation in GRAMD4‐overexpressing cells (Figure 5D). We next investigated whether GRAMD4 could promote the degradation of TAK1 by affecting its ubiquitination. We immunoprecipitated endogenous TAK1 with an antibody against TAK1 and probed the immunoblot for ubiquitin. HLF cells overexpressing GRAMD4 showed increased ubiquitination of TAK1 (Figure 5E). Further analysis showed that GRAMD4 increased the Lys48‐linked but not Lys63‐linked polyubiquitination of TAK1 (Figure 5F), and K48 ubiquitination is related to degradation by the proteasome.^31^ Taken together, these findings suggested that GRAMD4 promoted TAK1 degradation via Lys48‐linked polyubiquitination.

### 3.6 GRAMD4 recruited the E3 ligase ITCH to target TAK1 for Lys48‐ ubiquitination and degradation

**FIGURE 6:**
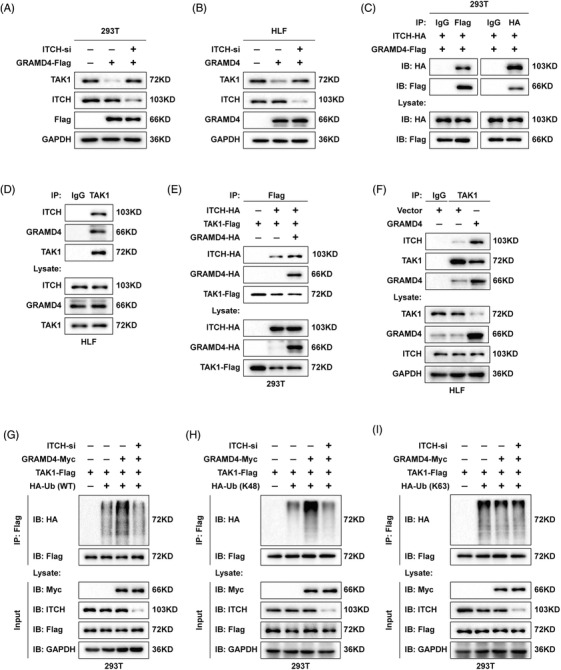
GRAMD4 recruited the E3 ligase ITCH to target TAK1 for K48‐linked ubiquitination and degradation. (A) 293T cells were transfected with vector or GRAMD4‐Flag plasmid together with control‐siRNA or ITCH‐siRNAs and analysed for TAK1, ITCH and Flag‐tag expression by western blot. (B) HLF cells with vector and GRAMD4 overexpression were transiently transfected with control‐siRNA or ITCH‐siRNAs and analysed for TAK1, ITCH and GRAMD4 expression by western blot. (C) Co‐IP analysis of the binding between exogenous GRAMD4‐Flag and ITCH‐HA in co‐transfected HEK293T cells. (D) Immunoassay of lysates from HLF cells, followed by immunoprecipitation with an anti‐TAK1 antibody. Co‐IP assay of binding between TAK1 and ITCH was performed at exogenous (E) endogenous (F) levels when GRAMD4 was overexpressed or not. Immunoassay of 293T cells transfected with expression vectors for various combinations of AMD4‐Myc, TAK1‐Flag, HA‐Ub (WT)(G), Ub (K48)(H) and Ub (K63)(I) together with control‐siRNA or ITCH‐siRNA, followed by immunoprecipitation of lysates with an anti‐Flag antibody and immunoblot analysis with anti‐Myc, anti‐HA, anti‐Flag anti‐ITCH antibodies

It was reported that ITCH is a specific E3 ligase of TAK1 that mediates its Lys48‐polyubiquitination,^32^ and we speculated that GRAMD4 might mediate the Lys48‐polyubiquitination of TAK1 in an ITCH‐dependent manner. To determine whether ITCH mediated the downregulation of TAK1 by GRAMD4, HEK‐293T cells were transfected with an siRNA targeting ITCH and a GRAMD4‐Flag expression plasmid, followed by determination of TAK1 expression. ITCH silencing rescued the decrease of TAK1 expression in 293T cells with GRAMD4 overexpression (Figure 6A). Similarly, the depletion of ITCH blocked the TAK1 depletion caused by GRAMD4 overexpression in HLF cells (Figure 6B). Subsequently, we performed co‐IP analysis with cell lysates of HEK‐293T cells transiently overexpressing GRAMD4‐Flag and ITCH‐HA, and the results showed that GRAMD4 interacted with ITCH (Figure 6C). Further experiments suggested that the *N*‐terminal region of GRAMD4 (aa 1–200) was required for its interaction with ITCH (Supporting information Figure S7A). Next, we investigated whether GRAMD4, ITCH and TAK1 co‐interacted in HLF cells. As shown in Figure 6D, these three proteins exhibited a heterotrimeric association. Moreover, the physical interaction between ITCH and TAK1 was enhanced when GRAMD4 was overexpressed in HEK‐293T and HLF cells (Figure 6E and F). Based on the results shown in Figure 3G, the amino acids 200–400 of GRAMD4 were required for its interaction with TAK1, and we proposed that GRAMD4 functions as a scaffold protein to recruit ITCH through its *N*‐terminus to TAK1 and promote their interaction.

These results demonstrated that GRAMD4 recruited ITCH to associate with TAK1. Since ITCH is a specific E3 ubiquitin ligase targeting TAK1, we investigated whether ITCH‐mediated TAK1 ubiquitination was enhanced by GRAMD4 overexpression. To test this, 293T cells were co‐transfected with vectors expressing GRAMD4‐Myc, TAK1‐Flag and HA‐ubiquitin (K48 or K63 linked HA‐Ub) combined with control‐siRNA or TAK1‐siRNA. The increase of TAK1 ubiquitination caused by GRAMD4 overexpression was abrogated by the downregulation of ITCH (Figure 6G, Supporting information Figure 7B). Similarly, the Lys48‐polyubiquitination of TAK1 was also decreased following the depletion of ITCH (Figure 6H, Supporting information Figure S7C). However, the Lys63‐polyubiquitination was not affected by GRAMD4 or ITCH (Figure 6I, Supporting information Figure S7D). Taken together, these data suggest that GRAMD4 recruits the E3 ligase ITCH to associate with TAK1, which in turn, induces the Lys48‐ubiquitination and degradation of TAK1.

### 3.7 GRAMD4 and TAK1 levels exhibited an inverse correlation in HCC tissues

**FIGURE 7:**
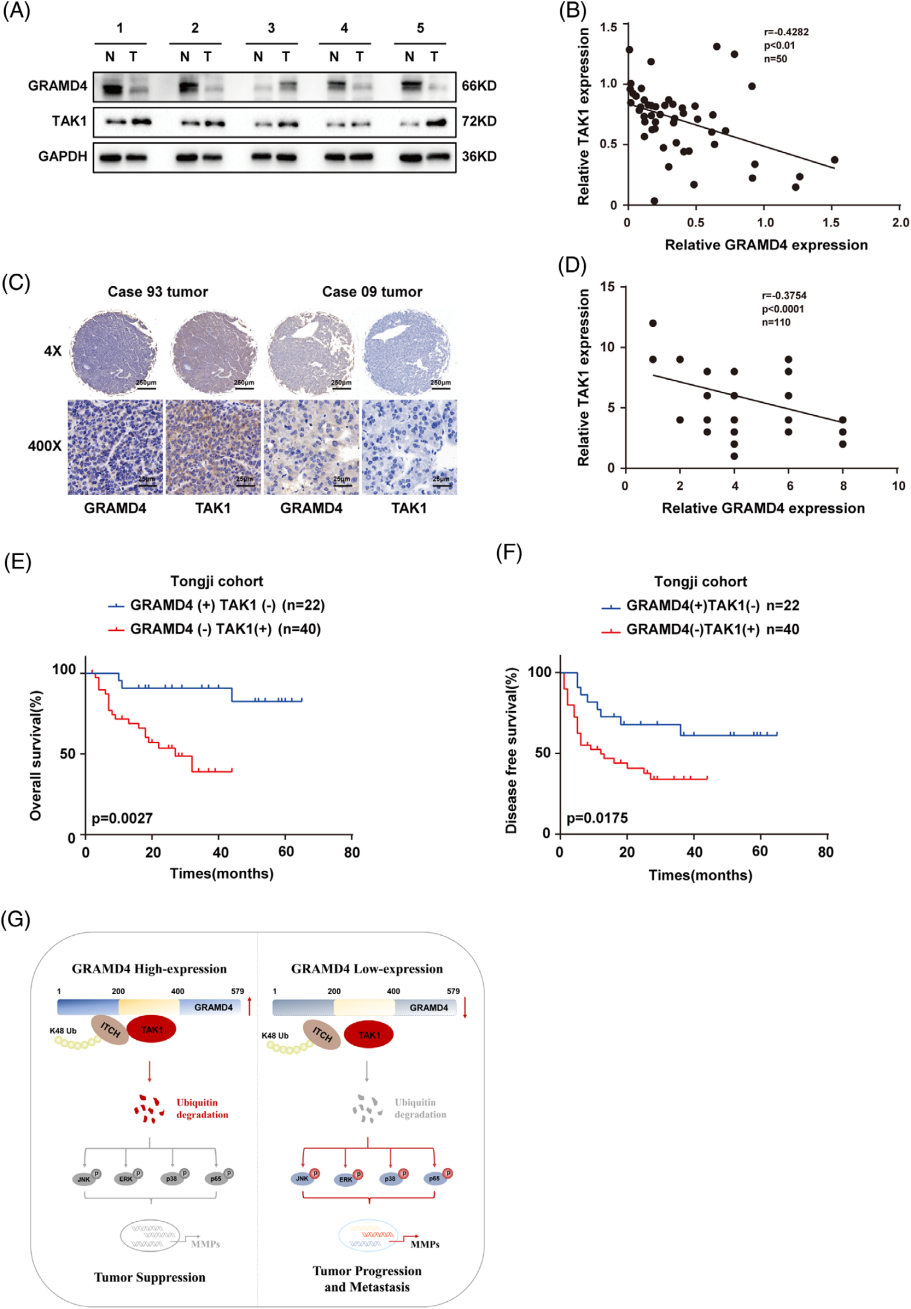
GRAMD4 and TAK1 had an inverse correlation in HCC tissues. (A) Western blot analysis of GRAMD4 and TAK1 expression in HCC and non‐cancerous tissues, GAPDH was used as a loading control. (B) Correlation analysis of GRAMD4 and TAK1 expression according to western blot analysis in HCC tissues from the investigated HCC patients. (C) IHC staining of GRAMD4 and TAK1 expression in HCC tissues from clinical HCC samples. The images are representative of figures from the investigated HCC patients (Scale bar: 250 μm, 25 μm). (D) Correlation analysis of GRAMD4 and TAK1 expression according to IHC staining in HCC tissues from the investigated HCC patients. (E‐F) Kaplan–Meier analysis of the overall and disease‐free survival of HCC patients stratified by GRAMD4/TAK1 expression levels. Statistical analysis was performed using Pearson's correlation in (B, D). (G) A proposed model for the mechanism through which GRAMD4 downregulates TAK1 by recruiting ITCH, resulting in the deactivation of the MAPK signalling pathway and inhibition of HCC progression and metastasis

To explore whether there is a correlation between the expression of GRAMD4 and TAK1 in HCC, GRAMD4 and TAK1 protein levels were evaluated by western blot analysis in 50 pairs of HCC samples and adjacent normal tissues. The results revealed that GRAMD4 expression in the HCC tissues were obviously lower than in the normal tissues. Conversely, TAK1 expression was higher in the tumour tissues than in adjacent normal tissues (Figure 7A, Supporting information Figure 8A and B). Moreover, correlation analysis revealed that GRAMD4 protein abundance was negatively correlated with TAK1 in the HCC tumour samples (r = −0.4282, *p* < 0.01, Pearson correlation, Figure 7B). In addition, IHC was used to analyse the expression of GRAMD4 and TAK1 in a tissue microarray containing 110 pairs of HCC samples and adjacent normal tissues. As predicted, TAK1 expression was higher in the tumour tissues than in the adjacent non‐tumour liver tissues (Supporting information Figure S8C and D), and it was negatively correlated with GRAMD4 (r = −0.3754, *p* < 0.0001, Pearson correlation, Figure 7C and D). Furthermore, we explored the protein abundance of GRAMD4 in other tumour types, as well as the correlation between the protein abundance of TAK1 and that of GRAMD4. The proteomics data used in this study were generated by the CPTAC, and the results of our analyses implied that the protein abundance of GRAMD4 was lower in the tumour tissues of COAD (colon adenocarcinoma), HNSC (head and neck squamous cell carcinoma), LIHC (liver hepatocellular carcinoma), LUSC (lung squamous cell carcinoma) and OV (ovarian serous cystadenocarcinoma), but it was elevated in the tumour tissues of KIRC (kidney renal clear cell carcinoma), LUAD (lung adenocarcinoma), PAAD (pancreatic adenocarcinoma) and UCEC (uterine corpus endometrial carcinoma) (Supporting information Figures S8E). In addition, TAK1 protein expression was negatively correlated with GRAMD4 in GBM (glioblastoma multiforme), LIHC, PAAD and UCEC (Supporting information Figure S8F). Furthermore, patients with low GRAMD4 expression combined with high TAK1 expression had shorter OS and DFS than patients with high GRAMD4 expression combined with low TAK1 expression (Figure 7E and F). These findings further support the notion that the expression of GRAMD4 was negatively associated with TAK1 in HCC.

In general, our study revealed a novel mechanism of TAK1 stability regulation mediated by GRAMD4 (Figure 7G). GRAMD4 inhibited HCC invasion and metastasis by recruiting ITCH to promote the Lys48‐ubiquitination and proteasomal degradation of TAK1. TAK1 degradation downregulated the MAPK pathway, which in turn, inhibited the expression of migration‐related genes, thereby, inhibiting the migration, invasion and metastasis of HCC.

## 4 DISCUSSION

GRAMD4 is a newly identified death‐inducing protein (DIP) that is associated with apoptosis in H1299, Saos‐2 cells and HCT 116 cells. However, there are few studies on the role of GRAMD4 in tumours. According to previous studies, the expression of GRAMD4 was elevated in lung squamous cell carcinoma, and high GRAMD4 expression predicted poor clinical outcomes. In our research, the proteomics data implied that the protein abundance of GRAMD4 was lower in the tumour tissues of COAD, HNSC, LIHC, LUSC and OV, but it was elevated in the tumour tissues of KIRC, LUAD, PAAD and UCEC. Furthermore, we studied the roles of GRAMD4 in HCC. According to our results, GRADM4 expression was decreased in HCC tissues, and patients with lower GRAMD4 expression had a poorer prognosis. Further univariate and multivariate Cox regression analyses revealed that GRAMD4 was an independent predictive factor for the prognosis of HCC patients. Further results showed that GRAMD4 suppressed the migration, invasion and motility of HCC cells in vitro, as well as repressing HCC metastasis in vivo.

Subsequently, we identified TAK1 as an interaction partner of GRAMD4 using LC‐MS/MS analysis. TAK1 is an intracellular serine/threonine kinase that regulates both NF‐κB and mitogen‐activated protein kinase (MAPK) signalling pathways, which are involved in diverse biological processes.^33^, ^34^ Our results showed that GRAMD4 could interact with TAK1, and the transmembrane domains (TMRs) of GRAMD4 were responsible for the interaction. The TMR‐truncation mutants GRAMD4‐M2 and GRAMD4‐M3 exhibited decreased binding to TAK1‐HA when co‐transfected with TAK1, similarly to the full‐length GRAMD4. However, it remains to be determined whether the TMRs are critical for the GRAMD4‐induced degradation of TAK1. TAK1 plays a dual role in tumour initiation, progression and metastasis. Hepatocyte‐specific TAK1‐deficient mice exhibited spontaneous hepatocyte death, inflammatory cell infiltration and carcinogenesis.^35^, ^36^ However, other studies found that TAK1 was upregulated during the development of several cancer types including HCC.^9^, ^37^, ^38^, ^39^

Our study demonstrated that GRAMD4 promoted the degradation of TAK1 and inhibited the activation of the MAPK and NF‐κB pathways. Furthermore, elimination of TAK1 expression by siRNA abrogated the inhibitory effects of GRAMD4 on the MAPK and NF‐κB pathways as well as the progression of HCC. Downstream signalling partners of TAK1 are regulated by TAK1 through dynamic polyubiquitination.^40^ A previous study revealed that USP4 is a deubiquitinase of TAK1 that could inhibit NF‐κB activation induced by TNF‐α and IL‐1β.^41^ Furthermore, Lys48‐polyubiquitination and subsequent degradation of TAK1 is crucial for the termination of NF‐κB activation induced by TNF‐α.^15^ Here, we found that GRAMD4 reduced TAK1 protein levels by promoting its Lys48‐polyubiquitination, suggesting that there is additional regulation of TAK1 stability via the ubiquitin‐proteasome pathway. However, as GRAMD4 is not an E3 ubiquitin ligase, additional E3 ligases must be involved in this process.

ITCH was previously found to be a specific E3 ligase targeting TAK1.^16^ ITCH belongs to the HECT E3 ubiquitin ligase family, which has been reported to act together with various adaptor proteins and contribute to the recruitment and subcellular localization of their substrates.^42^, ^43^ For example, TAX1BP1 can function as an adaptor to recruit ITCH to MAVS.^44^ In addition, PCBP2 was found to recruit MAVS to ITCH, which enhanced the degradation of MAVS.^45^ Here, we found that ITCH mediated the GRAMD4‐induced Lys48‐polyubiquitination of TAK1. Our results showed that GRAMD4 interacts with ITCH via the *N*‐terminal region (aa 1–200), while it interacts with TAK1 via the region encompassing amino acids 200–400 (containing two transmembrane domains). Moreover, GRAMD4 overexpression enhanced the interaction between TAK1 and ITCH, increasing the degradation of TAK1. These data suggest that GRAMD4 functions as an adaptor that recruits ITCH to TAK1. Our study, therefore, adds GRAMD4 to the growing list of ITCH adaptors.

In general, we revealed a new effect of GRAMD4 on the motility and metastasis of HCC cells, revealing how GRAMD4‐TAK1 interaction impaired the stability of TAK1 to decrease the activation of MAPK and NF‐κB signalling, which suppressed the motility and lung metastasis of HCC cells. Consequently, targeting GRAMD4 or modulating the GRAMD4‐TAK1 interaction may provide a promising therapeutic approach for the prevention of HCC metastasis.

## CONFLICT OF INTEREST

The authors declare that they have no conflict of interest.

## AUTHOR CONTRIBUTIONS

Z.B.X., Z.X.W. and D.Z.Y. conceptualized the study. G.Q.Y., C.J. and L.G.X. conducted most of the experiments. T.X.L., S.J., N.D., L.Q.M. and L.H.F. provided human specimens, clinical information and data analysis. G.Q.Y., Z.B.X., Z.X.W. and D.Z.Y. wrote the manuscript.

## Supporting information

Supporting Information

Supporting Information

Supporting Information

Supporting Information

Supporting Information

Supporting Information

Supporting Information

Supporting Information

Supporting Information

Supporting Information

Supporting Information

## Data Availability

The data that support the findings of this study are available from the corresponding author upon reasonable request.
