# Supplementary figures and images for: GRAMD4 inhibits tumour metastasis by recruiting the E3 ligase ITCH to target TAK1 for degradation in hepatocellular carcinoma
Source: Clin Transl Med. 2021 Nov 17;11(11):e635. doi: 10.1002/ctm2.635 (PMC8597946; doi:10.1002/ctm2.635)

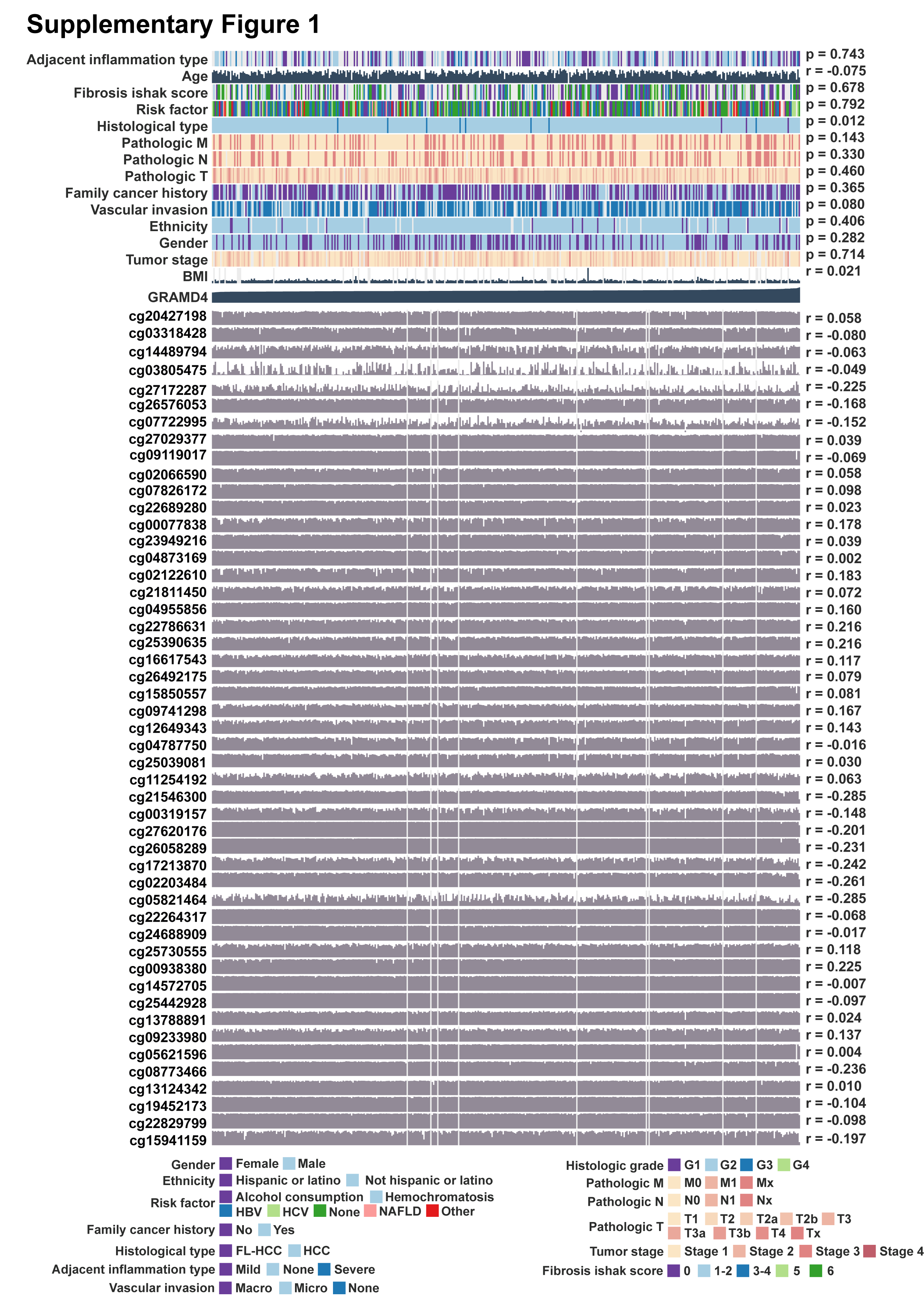

Supplement: Supplementary file 1 — Supporting Information [file CTM2-11-e635-s010.tif]

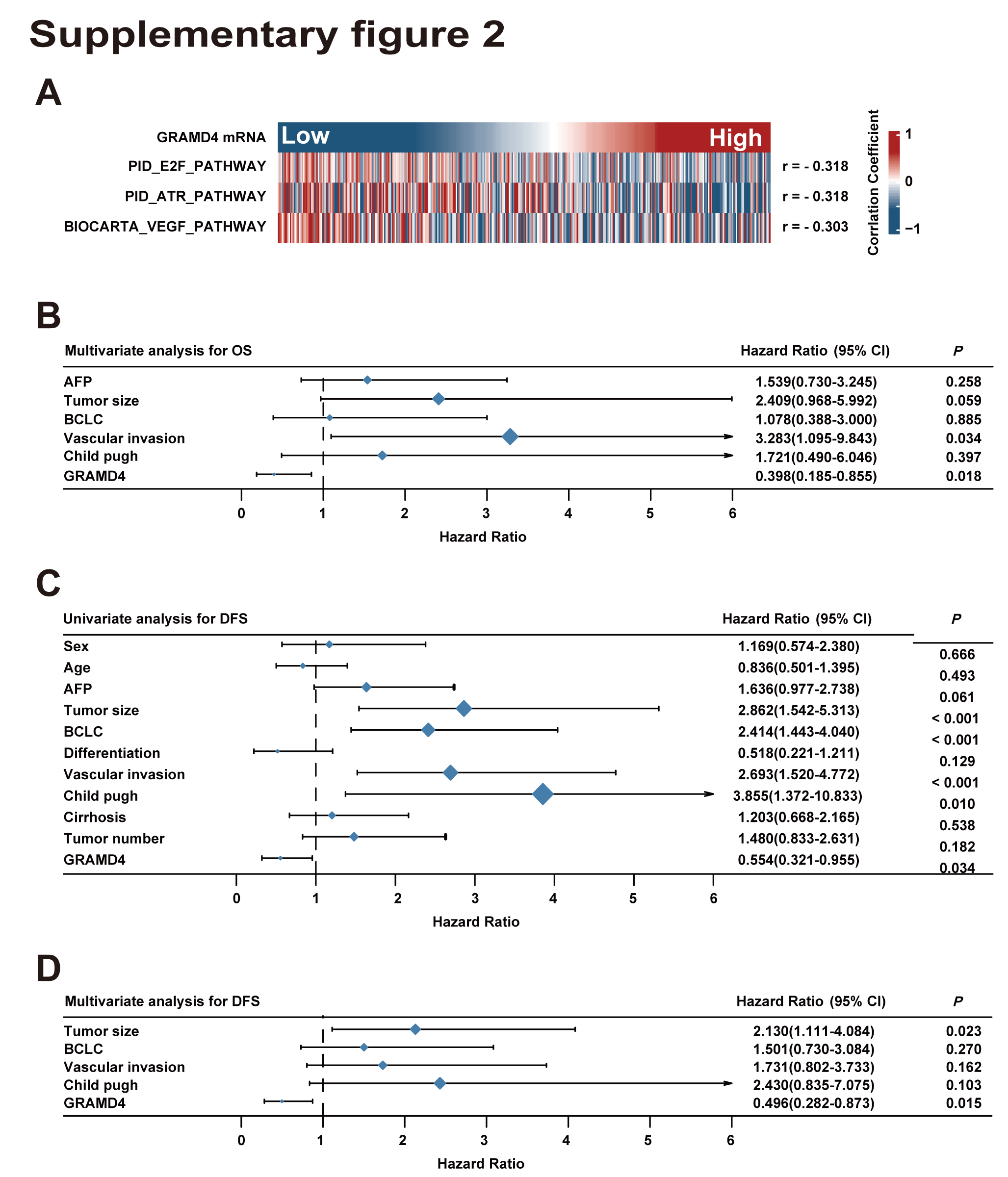

Supplement: Supplementary file 2 — Supporting Information [file CTM2-11-e635-s006.tif]

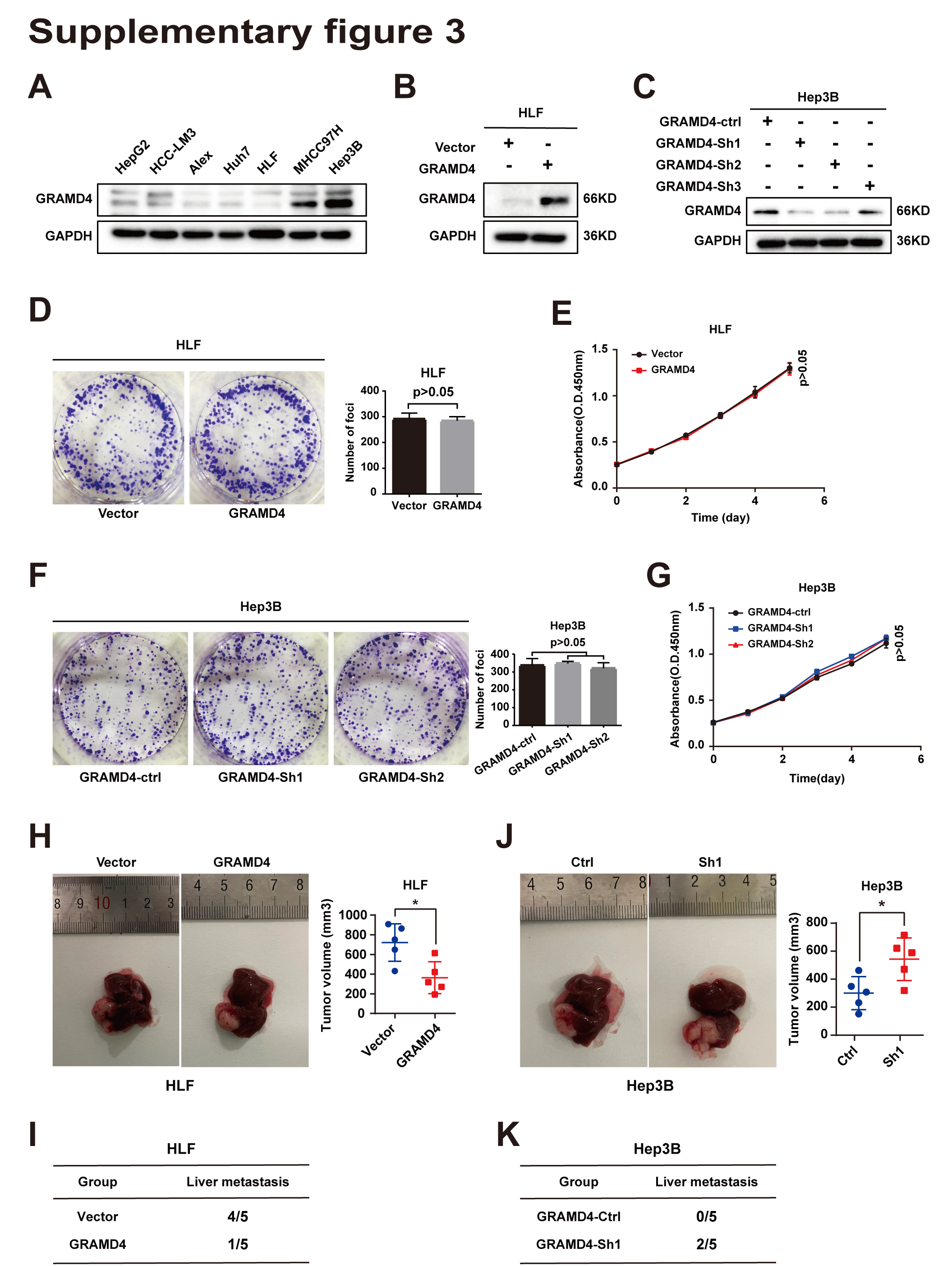

Supplement: Supplementary file 3 — Supporting Information [file CTM2-11-e635-s002.tif]

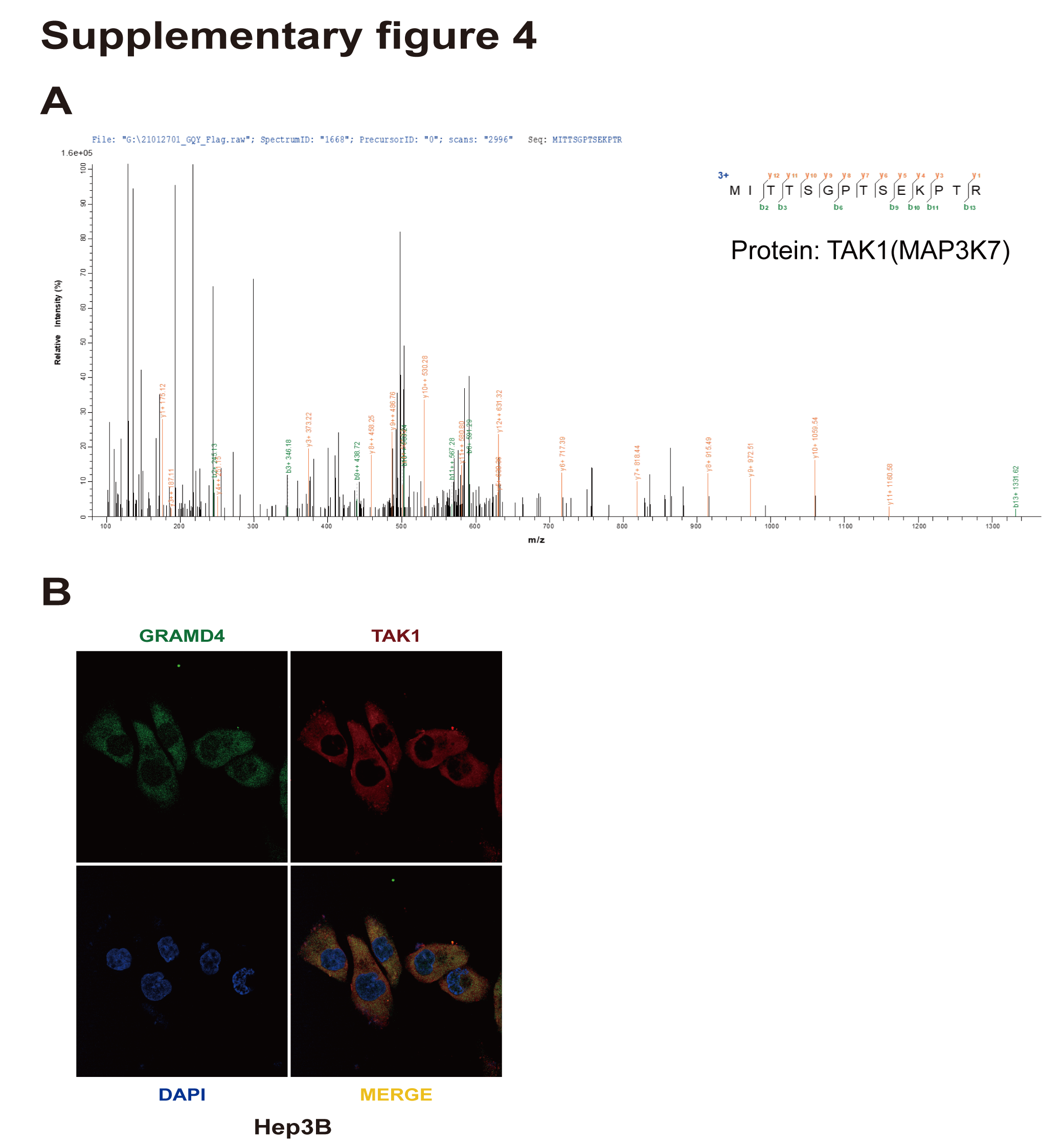

Supplement: Supplementary file 4 — Supporting Information [file CTM2-11-e635-s003.tif]

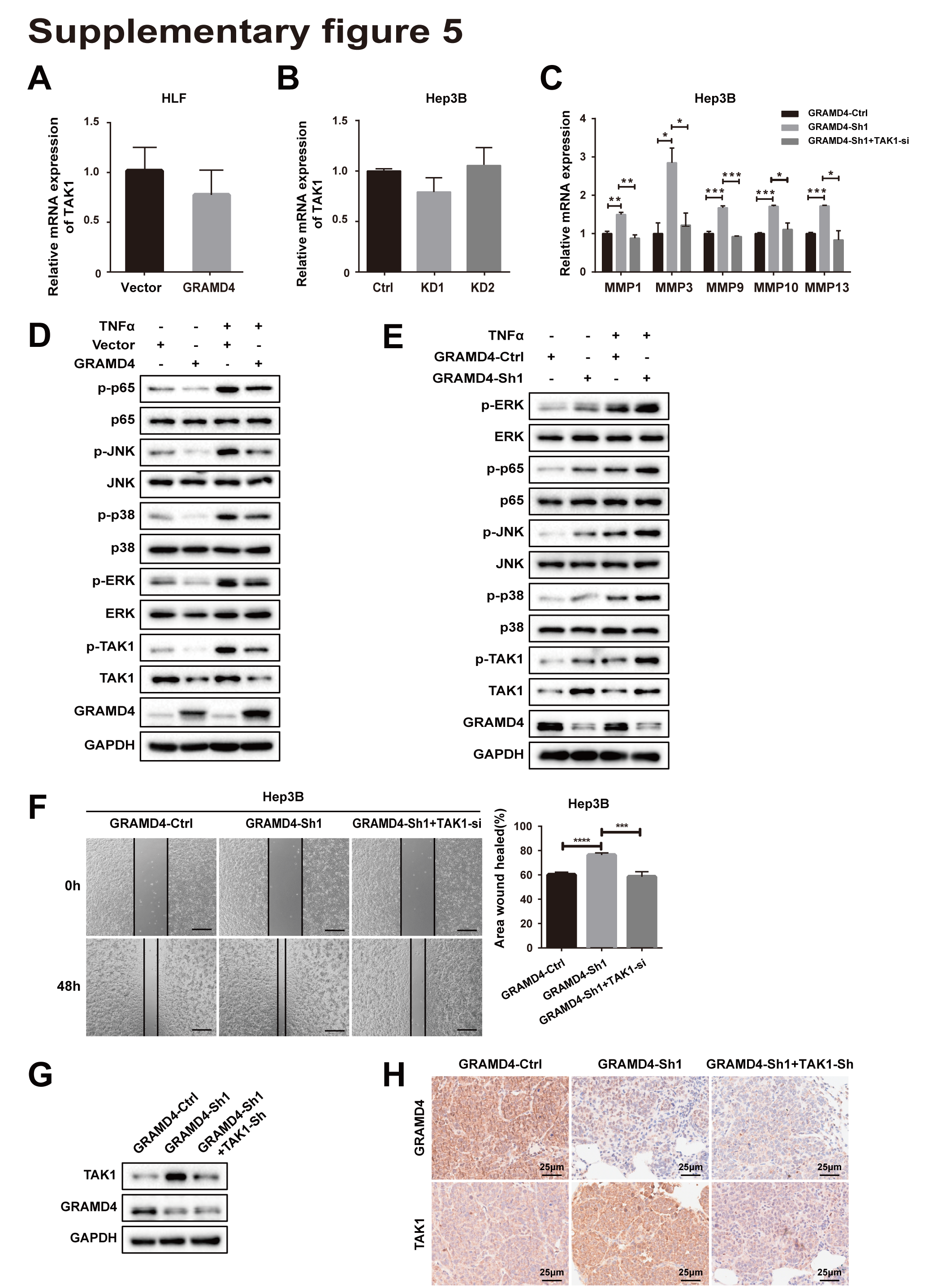

Supplement: Supplementary file 5 — Supporting Information [file CTM2-11-e635-s008.tif]

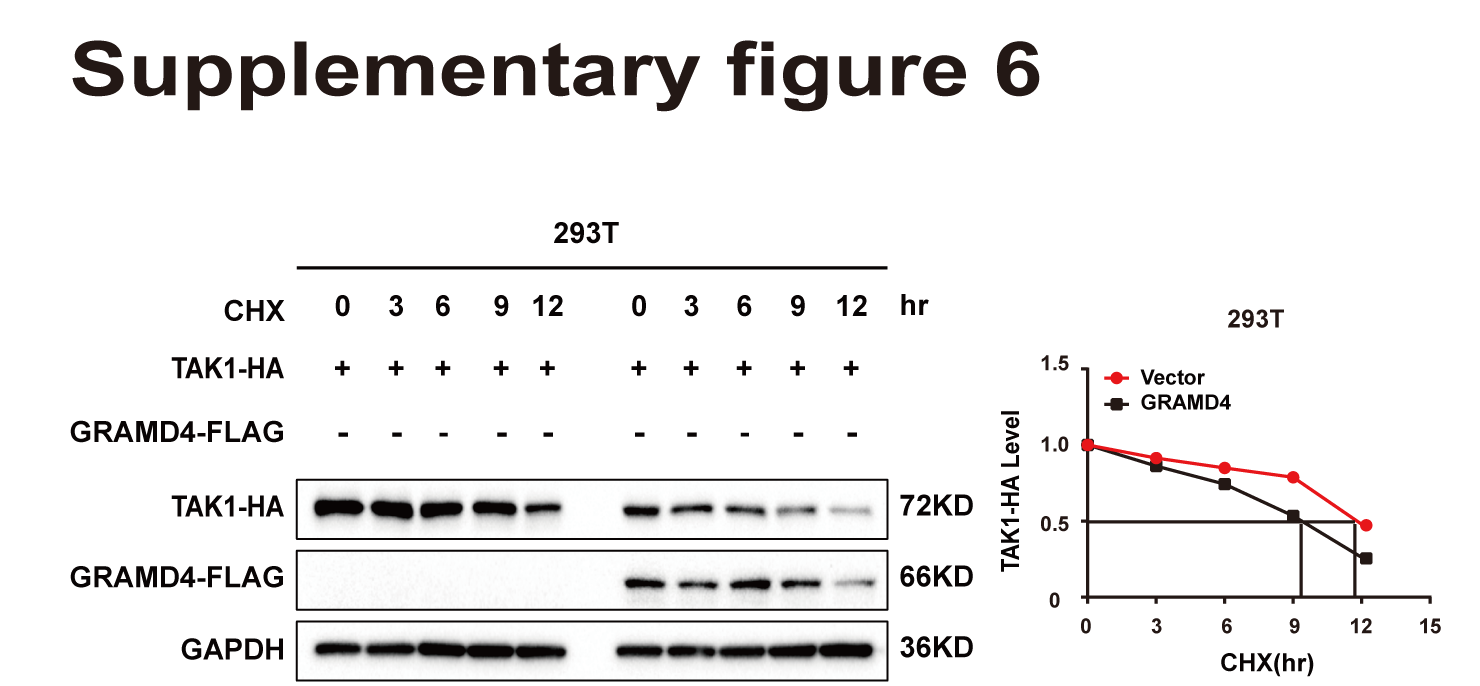

Supplement: Supplementary file 6 — Supporting Information [file CTM2-11-e635-s001.tif]

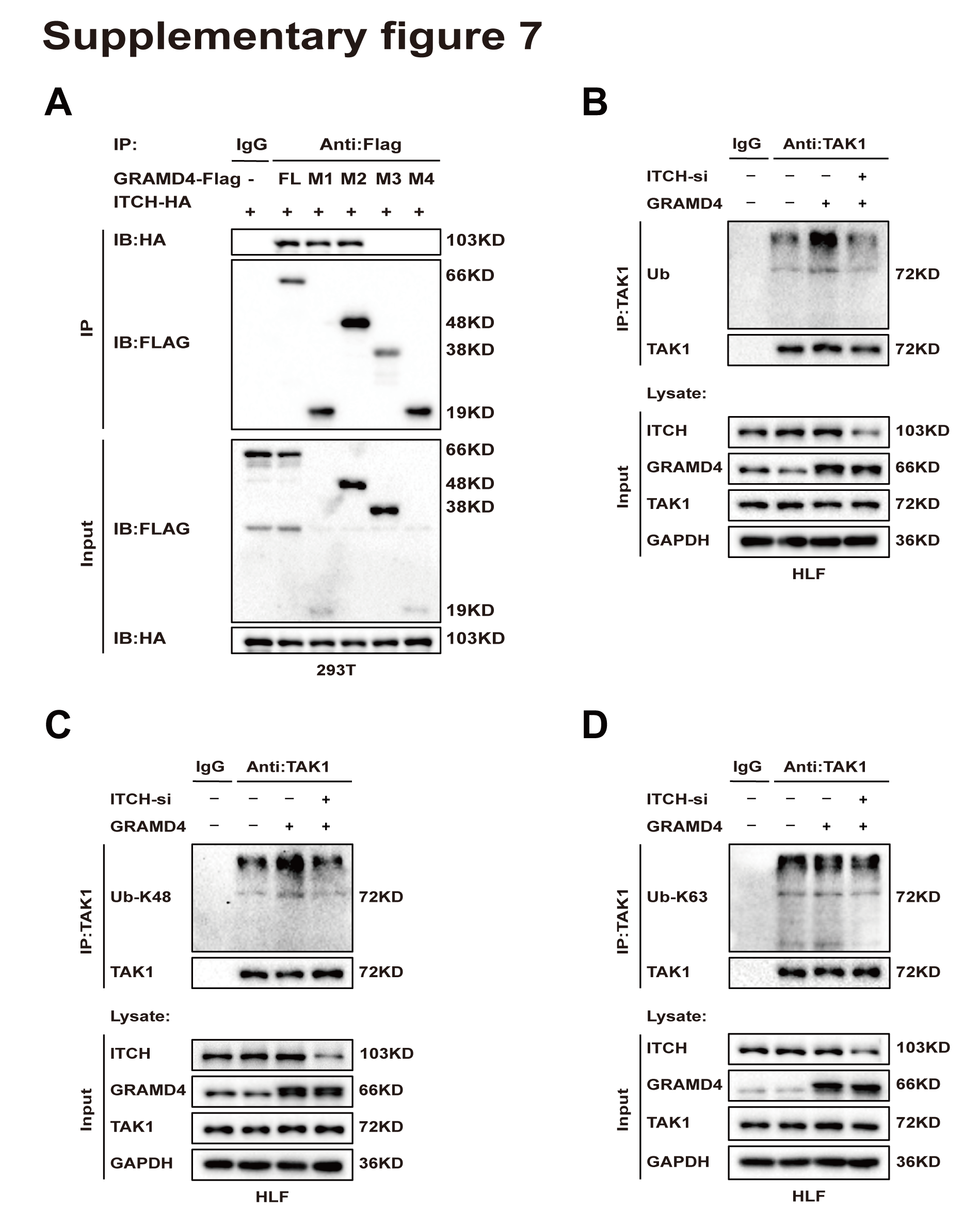

Supplement: Supplementary file 7 — Supporting Information [file CTM2-11-e635-s011.tif]

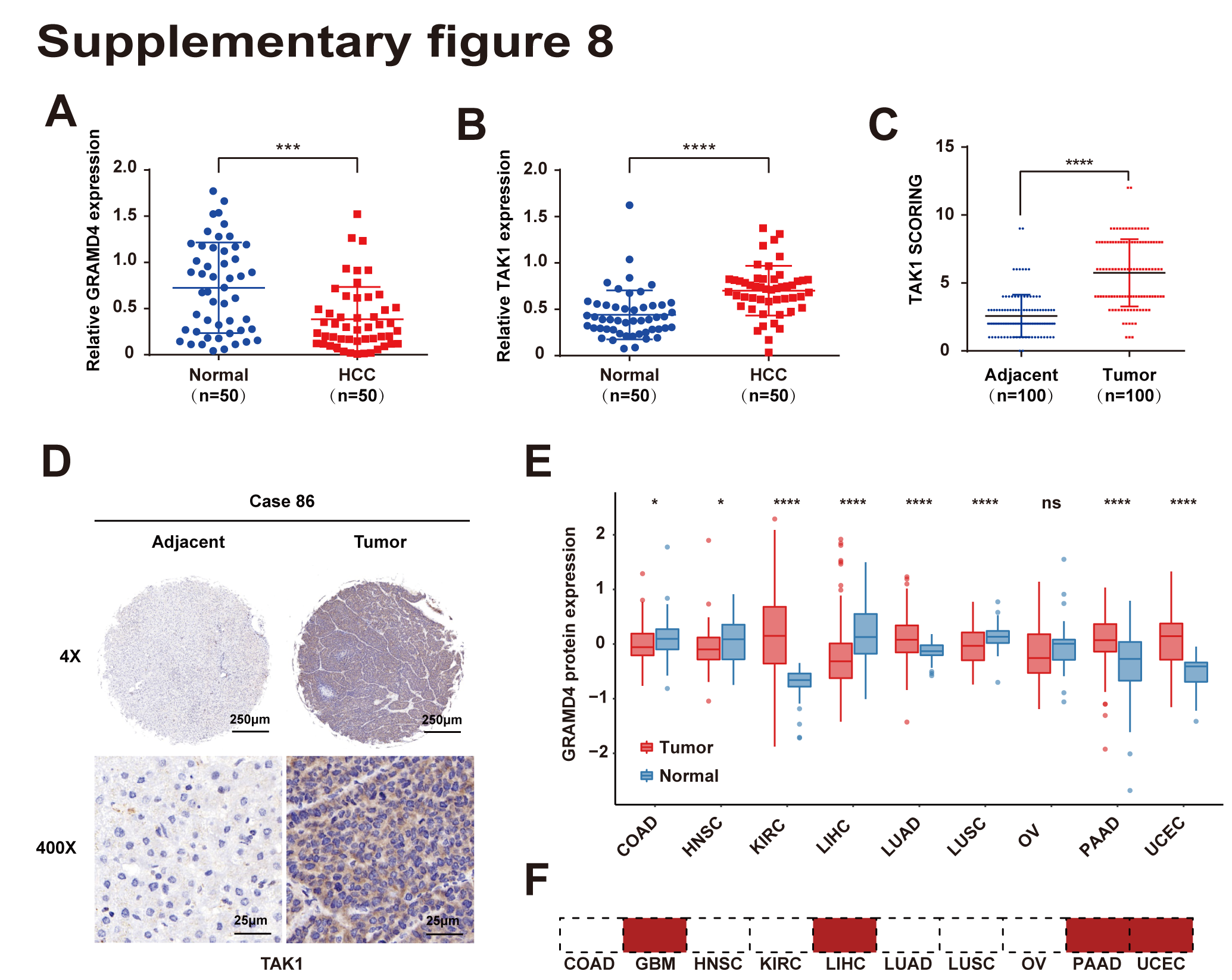

Supplement: Supplementary file 8 — Supporting Information [file CTM2-11-e635-s005.tif]
