# Supplementary material for: GRAMD4 inhibits tumour metastasis by recruiting the E3 ligase ITCH to target TAK1 for degradation in hepatocellular carcinoma
Source: Clin Transl Med. 2021 Nov 17;11(11):e635. doi: 10.1002/ctm2.635 (PMC8597946; doi:10.1002/ctm2.635)
Supplement: Supplementary file 9 — Supporting Information [file CTM2-11-e635-s009.docx]

**Supplementary Figure 1.** Correlation between the expression level of GRAMD4 and methylation levels of corresponding CpG sites. The coefficients were calculated by Pearson's correlation analysis.

**Supplementary Figure 2.** (A) Multivariate regression analysis of the correlation between the GRAMD4 and clinicopathological characteristics regarding OS. Univariate (B) and multivariate (C) regression analysis of the correlation between GRAMD4 and clinicopathological characteristics regarding DFS in the Tongji cohort.

**Supplementary Figure 3.** GRAMD4 protein expression in 7 selected HCC cell lines was examined by western blot analysis (A). (B) HLF cells were transfected with lentivirus-GRAMD4, and stable overexpression of GRAMD4 in HLF cells was confirmed. (C) Hep3B cells were transfected with three shRNAs against GRAMD4 and ctrl shRNAs, stable knockdown of GRAMD4 in Hep3B cells were confirmed. (D) HLF-GRAMD4 cell proliferation was compared with corresponding vector control cells using the CCK-8 assay. (E) Cell proliferation was examined using a colony formation assay, and the relative clone number was used for statistical analysis. Data represent the means ± SD of three independent experiments. (F) Cell proliferation was measured using the CCK-8 assay. (G) Cell proliferation was examined using a colony formation assay, and the relative clone number was used for statistical analysis. Data represent the means ± SD of three independent experiments. (H, J) Representative images of liver tissues from the orthotopic mouse model.

(I, K) The incidence of liver metastasis in the orthotopic model mice was calculated as shown in the panels.

**Supplementary Figure 4.** (A) Collision-induced dissociation spectra of TAK1 peptides identified using gradient elution LC-MS/MS analysis of the binding proteins from FLAG-GRAMD4 immunoprecipitation. (B) Hep3B cells were fixed and stained with TAK1 antibody (red) and GRAMD4 antibody (green). Nuclei were counter-stained with DAPI (blue).

**Supplementary Figure 5.** (A-B) The expression levels of TAK1 were analyzed by qRT-PCR in the indicated cells. (C) The expression levels of MMP-1, MMP-3, MMP-9, MMP-10 and MMP-13 were analyzed by qRT-PCR in the indicated cells. (D) Western blot analysis of TAK1, p-JNK, p-p38, p-ERK and p-ERK p65 protein levels in HLF cells stably transfected with GRAMD4 plasmid or vector control. (E) Western blot analysis of TAK1, p-JNK, p-p38, p-ERK and p-ERK 65 protein levels in Hep3B cells stably transfected with lentivirus expressing Sh-Ctrl, GRAMD4-Sh1or GRAMD4-Sh2. (F) Wound healing assays were performed with the indicated cells. Experiments were performed in triplicate and the data are shown as means ± SD. GRAMD4-knockdown Hep3B cells were transfected with shRNA targeting TAK1, stable knockdown of TAK1 in Hep3B cells was confirmed by western blot (G) and IHC (H).

**Supplementary Figure 6.** 293T cells transfected with GRAMD4-Flag plasmid or vector control and TAK1-HA were cultured for 36h before being further incubated with CHX (20μg/mL) for 0, 4, 8, and 12 h. The TAK1-HA protein levels of the transfected cells were determined by western blot analysis.

**Supplementary Figure 7.** (A) Co-IP analysis of the interaction between TAK1 and GRAMD4 or GRAMD4 truncation mutants in HEK293T cells co-transfected with TAK1-HA plasmid together with GRAMD4-Flag plasmid or GRAMD4-Flag truncation mutant plasmids. Immunoassay of vector control and GRAMD4 overexpressing-HLF cells transfected with control-siRNA or ITCH-siRNA, followed by immunoprecipitation of lysates with an anti-TAK1 antibody and immunoblot analysis with anti-Ub (WT) (B), anti-Ub (K48) (C), anti-Ub (K63) (D), and anti-TAK1 antibodies.

**Supplementary Figure 8.** Statistical analysis of GRAMD4 (A) and TAK1 (B) expression according to western blot analysis in 50 paired HCC tumor samples and adjacent non-tumor tissues. (C) Statistical analysis of TAK1 expression according to the IHC assay in unpaired HCC tissues and matched adjacent tissues. (D). Representative immunohistochemical staining images of TAK1 in 110 paired HCC tumor samples and adjacent non-tumor tissues (Scale bars: 250 μm, 25 μm). (E) Protein abundance of GRAMD4 in tumor samples and matched normal tissues across cancer types. (F) Pearson's correlation between GRAMD4 and TAK1 protein abundance across cancer types. COAD (Colon adenocarcinoma), HNSC (Head and Neck squamous cell carcinoma), KIRC (Kidney renal clear cell carcinoma), LIHC (Liver hepatocellular carcinoma), LUAD (Lung adenocarcinoma),LUSC (Lung squamous cell carcinoma), OV (Ovarian serous cystadenocarcinoma), PAAD (Pancreatic adenocarcinoma) and UCEC (Uterine Corpus Endometrial Carcinoma), GBM (Glioblastoma multiforme)
