# Supplementary material for: GRAMD4 inhibits tumour metastasis by recruiting the E3 ligase ITCH to target TAK1 for degradation in hepatocellular carcinoma
Source: Clin Transl Med. 2021 Nov 17;11(11):e635. doi: 10.1002/ctm2.635 (PMC8597946; doi:10.1002/ctm2.635)
Supplement: Supplementary file 10 — Supporting Information [file CTM2-11-e635-s004.docx]

**Supplementary Table 1. Sequence of primers for qRT-PCR**

| **Gene** | **Forward (5’ to 3’)** | **Reverse (5’ to 3’)** |
| --- | --- | --- |
| **GRAMD4** | ACTTCCTCGATCTAGCGGAGT | TCTGTCCGGTTAAAGTCCTGG |
| **TAK1** | AACTGCTTCCTGTATGGGGTC | AAGGCGTCGTCAATGGACTC |
| **MMP1** | ACTCTGGAGTAATGTCACACCT | GTTGGTCCACCTTTCATCTTCA |
| **MMP3** | AGTCTTCCAATCCTACTGTTGCT | TCCCCGTCACCTCCAATCC |
| **MMP9** | GGGACGCAGACATCGTCATC | TCGTCATCGTCGAAATGGGC |
| **MMP10** | CCCACTCTACAACTCATTCACAG | TCAGATCCCGAAGGAACAGAT |
| **MMP13** | ACTGAGAGGCTCCGAGAAATG | GAACCCCGCATCTTGGCTT |
| **GAPDH** | GGGGCTCTCCAGAACATCATCC | ACGCCTGCTTCACCACCTCTT |
