# Supplementary material for: GRAMD4 inhibits tumour metastasis by recruiting the E3 ligase ITCH to target TAK1 for degradation in hepatocellular carcinoma
Source: Clin Transl Med. 2021 Nov 17;11(11):e635. doi: 10.1002/ctm2.635 (PMC8597946; doi:10.1002/ctm2.635)
Supplement: Supplementary file 11 — Supporting Information [file CTM2-11-e635-s007.docx]

**Supplementary Table 2. Proteins Identified In LS-MS**

| **Accession** | **Gene** | **Flag** | **IgG** | **log2(Flag/IgG)** |
| --- | --- | --- | --- | --- |
| Q6IC98 | **GRAMD4** | 34.6012 | 24.1557 | 10.4455 |
| A0A0B4J1Z1 | SRSF7 | 24.5443 | 19.5076 | 5.0367 |
| P01859 | IGHG2 | 24.1545 | 19.1902 | 4.9643 |
| O43318 | **MAP3K7** | 23.3352 | 19.0161 | 4.3191 |
| P10412 | H1-4 | 23.1286 | 19.079 | 4.0496 |
| Q9H814 | PHAX | 24.5308 | 20.583 | 3.9478 |
| Q15434 | RBMS2 | 34.4728 | 30.69 | 3.7828 |
| P41091 | EIF2S3 | 22.4496 | 19.0571 | 3.3925 |
| Q9UBF2 | COPG2 | 24.2058 | 21.086 | 3.1198 |
| G3V3B0 | ACIN1 | 27.0554 | 23.9988 | 3.0566 |
| Q96E39 | RBMXL1 | 23.8749 | 21.1381 | 2.7368 |
| P04843 | RPN1 | 22.5496 | 19.9072 | 2.6424 |
| A0A494C1T2 | MTHFD1 | 24.5423 | 21.9327 | 2.6096 |
| I3L1P8 | SLC25A11 | 22.3942 | 19.7934 | 2.6008 |
| A0A2R8Y705 | POLD1 | 22.7312 | 20.1327 | 2.5985 |
| Q04837 | SSBP1 | 20.7451 | 18.1799 | 2.5652 |
| P42285 | MTREX | 24.5579 | 22.0109 | 2.547 |
| H0Y8X1 | SDHA | 22.6089 | 20.118 | 2.4909 |
| Q9NUD5 | ZCCHC3 | 29.2317 | 26.8309 | 2.4008 |
| P27694 | RPA1 | 21.7863 | 19.405 | 2.3813 |
| Q13263 | TRIM28 | 22.1414 | 19.7897 | 2.3517 |
| Q8NC60 | NOA1 | 23.1912 | 20.8605 | 2.3307 |
| Q8IXB1 | DNAJC10 | 23.8974 | 21.5832 | 2.3142 |
| P79522 | PRR3 | 22.7913 | 20.5237 | 2.2676 |
| A0A087WV29 | NAT10 | 22.9238 | 20.6972 | 2.2266 |
| B7Z645 | SYNCRIP | 26.7427 | 24.5476 | 2.1951 |
| O75534 | CSDE1 | 23.2687 | 21.0906 | 2.1781 |
| Q6UN15 | FIP1L1 | 22.9076 | 20.7734 | 2.1342 |
| C9J9K3 | RPSA | 23.0448 | 20.9441 | 2.1007 |
| A0A0A0MSI0 | PRDX1 | 20.8913 | 18.8232 | 2.0681 |
| O60832 | DKC1 | 27.5049 | 25.4562 | 2.0487 |
| A0A2R8YFS5 | DDX3X | 28.6488 | 26.6384 | 2.0104 |
